# Understanding How Clinicians Personalize Fluid and Vasopressor Decisions in Early Sepsis Management

**DOI:** 10.1001/jamanetworkopen.2024.7480

**Published:** 2024-04-19

**Authors:** Elizabeth S. Munroe, Julien Weinstein, Hayley B. Gershengorn, Kevin J. Karlic, Sarah Seelye, Michael W. Sjoding, Thomas S. Valley, Hallie C. Prescott

**Affiliations:** 1 Division of Pulmonary and Critical Care Medicine, Department of Medicine, University of Michigan, Ann Arbor; 2 VA Center for Clinical Management Research, Ann Arbor, Michigan; 3 Division of Pulmonary, Critical Care, and Sleep Medicine, University of Miami Miller School of Medicine, Miami, Florida; 4 Division of Critical Care Medicine, Albert Einstein College of Medicine, Bronx, New York; 5 Department of Medicine, University of Michigan, Ann Arbor

## Abstract

**Importance:**

Recent sepsis trials suggest that fluid-liberal vs fluid-restrictive resuscitation has similar outcomes. These trials used generalized approaches to resuscitation, and little is known about how clinicians personalize fluid and vasopressor administration in practice.

**Objective:**

To understand how clinicians personalize decisions about resuscitation in practice.

**Design, Setting, and Participants:**

This survey study of US clinicians in the Society of Critical Care Medicine membership roster was conducted from November 2022 to January 2023. Surveys contained 10 vignettes of patients with sepsis where pertinent clinical factors (eg, fluid received and volume status) were randomized. Respondents selected the next steps in management. Data analysis was conducted from February to September 2023.

**Exposure:**

Online Qualtrics clinical vignette survey.

**Main Outcomes and Measures:**

Using multivariable logistic regression, the associations of clinical factors with decisions about fluid administration, vasopressor initiation, and vasopressor route were tested. Results are presented as adjusted proportions with 95% CIs.

**Results:**

Among 11 203 invited clinicians, 550 (4.9%; 261 men [47.5%] and 192 women [34.9%]; 173 with >15 years of practice [31.5%]) completed at least 1 vignette and were included. A majority were physicians (337 respondents [61.3%]) and critical care trained (369 respondents [67.1%]). Fluid volume already received by a patient was associated with resuscitation decisions. After 1 L of fluid, an adjusted 82.5% (95% CI, 80.2%-84.8%) of respondents prescribed additional fluid and an adjusted 55.0% (95% CI, 51.9%-58.1%) initiated vasopressors. After 5 L of fluid, an adjusted 17.5% (95% CI, 15.1%-19.9%) of respondents prescribed more fluid while an adjusted 92.7% (95% CI, 91.1%-94.3%) initiated vasopressors. More respondents prescribed fluid when the patient examination found dry vs wet (ie, overloaded) volume status (adjusted proportion, 66.9% [95% CI, 62.5%-71.2%] vs adjusted proportion, 26.5% [95% CI, 22.3%-30.6%]). Medical history, respiratory status, lactate trend, and acute kidney injury had small associations with fluid and vasopressor decisions. In 1023 of 1127 vignettes (90.8%) where the patient did not have central access, respondents were willing to start vasopressors through a peripheral intravenous catheter. In cases where patients were already receiving peripheral norepinephrine, respondents were more likely to place a central line at higher norepinephrine doses of 0.5 µg/kg/min (adjusted proportion, 78.0%; 95% CI, 74.7%-81.2%) vs 0.08 µg/kg/min (adjusted proportion, 25.2%; 95% CI, 21.8%-28.5%) and after 24 hours (adjusted proportion, 59.5%; 95% CI, 56.6%-62.5%) vs 8 hours (adjusted proportion, 47.1%; 95% CI, 44.0%-50.1%).

**Conclusions and Relevance:**

These findings suggest that fluid volume received is the predominant factor associated with ongoing fluid and vasopressor decisions, outweighing many other clinical factors. Peripheral vasopressor use is common. Future studies aimed at personalizing resuscitation must account for fluid volumes and should incorporate specific tools to help clinicians personalize resuscitation.

## Key Points

**Question:**

How do clinicians personalize decisions about fluid and vasopressor administration for patients in early stages of sepsis?

**Findings:**

In this survey study of 550 US critical care clinicians, respondents relied on fluid volume already received more than other clinical factors (eg, respiratory status or lactate trend) to inform decisions about fluid and vasopressor administration. Most respondents chose to start vasopressors peripherally, with vasopressor dose, trend, and duration associated with the subsequent decision to place a central line.

**Meaning:**

These findings suggest that fluid volume received is the predominant factor guiding ongoing fluid and vasopressor decisions. Future studies aimed at personalizing resuscitation in sepsis must account for fluid volume and should incorporate specific tools to help clinicians personalize care.

## Teaser

This survey study assesses factors associated with personalizing decision-making regarding fluid and vasopressor administration among patients with sepsis.

## Introduction

Sepsis is a major cause of morbidity and mortality, accounting for up to 1 in every 2 hospital deaths in the US.^1,2^ Patients who develop sepsis-induced hypotension are typically treated using intravenous (IV) fluids, with vasopressors reserved for refractory hypotension and shock.^3,4^ Traditional recommendations to administer vasopressors via central venous access have also historically limited how quickly vasopressors can be started. Because central lines take time to place, clinicians often rely on fluids to support blood pressure during central line placement.^5,6^ However, there has been recent concern that aggressive fluid resuscitation and fluid overload may increase organ failure and mortality in patients with sepsis.^7,8,9^ Concurrently, emerging data have demonstrated that administering vasopressors through peripheral IVs (PIVs) is safe, which may help facilitate earlier vasopressor initiation.^5,10,11,12,13^ As a result, there has been increasing interest in restricting fluid volumes and starting vasopressors earlier.^14,15^

To date, clinical trials comparing fluid-restrictive, early vasopressor treatment vs fluid-liberal strategies in patients with sepsis have yielded neutral results.^16,17^ However, these trials have used generalized protocols and broad clinical criteria, rather than personalized approaches, to guide resuscitation. Little is known about how clinicians make decisions about fluid and vasopressor administration in practice. Understanding existing resuscitation approaches and how clinicians personalize resuscitation decisions is important to informing future clinical trials aimed at optimizing sepsis resuscitation.

In this study, we sought to determine clinical factors associated with clinician decisions about fluid administration, vasopressor initiation, and vasopressor route in early sepsis using a randomized vignette survey method. This survey design has been demonstrated to elicit preferences and predict behavior, providing insight into clinician decision-making.^18,19,20^

## Methods

### Survey Development and Administration

This survey study was deemed exempt from review by the University of Michigan institutional review board because the study used an anonymous survey in accordance with the Common Rule. The study followed the American Association for Public Opinion Research (AAPOR) reporting guideline.^21^ We conducted a randomized vignette survey through the Society of Critical Care Medicine (SCCM). The survey was administered in Qualtrics. We piloted the survey with a group of critical care physician researchers. After incorporating feedback, we tested the updated survey using cognitive interviewing sessions and iterative modification with 7 additional critical care physicians and advanced practice clinicians (APPs; eg, nurse practitioners and physician assistants) not included in the final survey.

The final survey was distributed on November 30, 2022, via email through the SCCM member list-serve to US-based physicians and APPs (as determined based on internal SCCM demographic information). The first page of the survey provided information about informed consent. A participant was deemed to have consented to the study if they proceeded to the next page. Responses were anonymous. A reminder email was sent 3 weeks later. The survey closed after 7 weeks. Respondents who completed the survey were offered the opportunity to enter a raffle for twenty $50 gift cards.

### Survey Structure

The survey consisted of 10 clinical vignettes followed by questions about general practices and demographics. Vignettes presented patients with sepsis and hypotension and were divided into 2 sections: fluid and vasopressor management (cases 1-6) and central line placement (cases 7-10). Vignette order was randomized within each section. Each vignette included randomized clinical factors (eAppendix 1 in Supplement 1), which were selected based on literature review and clinical experience. Respondents had the option to comment on each case using a free-text response box. After completing all 10 vignettes, respondents were asked to assess case realism on a 4-point scale (unrealistic to very realistic) to self-report their usual sepsis resuscitation practices and to provide basic demographic information. The full survey is provided in eAppendix 2 in Supplement 1.

### Clinical Vignettes and Randomized Factors

Cases 1 to 6 assessed resuscitation practices and vasopressor initiation route. In each case, respondents were asked whether they would give additional fluid, start vasopressors, both, or neither. Respondents who chose to start vasopressors were asked what route they would use: PIV only, PIV as a bridge to a central venous catheter (CVC), or central access only (new CVC or preexisting central access, if applicable). Respondents were also asked to rate the difficulty of each case on a 5-point Likert scale.

Among cases 1 to 6, each set of 2 cases were paired (1 and 2, 3 and 4, and 5 and 6). Baseline vascular access was set in the question stem with each case pair having 1 case where the patient had only peripheral access (2 PIVs) and 1 case where the patient already had central access (temporary CVC, port, or peripherally inserted central catheter [PICC]). For all 6 cases, the amount of fluid the patient had already received (1 L, 2 L, or 5 L) and current mean arterial pressure (MAP; 52 mm Hg, 58 mm Hg, or 64 mm Hg) were randomized. Cases 1 and 2 included additional randomized factors related to an assessment of the patient’s volume status by clinical examination (dry, euvolemic, or wet) and past medical history (chronic obstructive pulmonary disease, heart failure with reduced ejection fraction [HFrEF], or kidney failure and receiving dialysis). Cases 3 and 4 included randomized supplemental oxygen support (room air, 6-L nasal cannula, or 50% face mask) and respiratory rate (20 breaths per minute, 30 breaths per minute, or 40 breaths per minute). Cases 5 and 6 included randomized laboratory-based factors: lactate trend after fluids (increasing, decreasing, or repeat pending) and acute kidney injury (AKI; no AKI, nonoliguric AKI, or oliguric AKI). Factors were individually randomized, so respondents could have theoretically seen the same conditions across cases (eg, 1 L of fluid in all cases 1-6, or dry examination in cases 1 and 2).

Cases 7 to 10 assessed clinician threshold for placing central access in patients with septic shock already receiving norepinephrine through an 18-gauge PIV. Respondents were asked whether they would continue norepinephrine through the PIV or place new central access. Vasopressor dose (0.08 μg/kg/min, 0.2 μg/kg/min, or 0.5 μg/kg/m), dose trend (decreasing, stable, or increasing), duration (8 hours or 24 hours), and PIV location (forearm or upper arm) were randomized for each case.

To ensure case realism, certain background characteristics (age, sex, and sepsis etiology) were provided but were not randomized. In cases 1 to 6, these fixed characteristics were similar within each case pair (eg, cases 1 and 2 presented patients with similar age and sex).

### Statistical Analysis

Respondents who completed at least 1 vignette were included in the analysis. Survey weighting was not used. Descriptive statistics were used to assess participant characteristics, usual practice, route of vasopressor initiation, and case realism. We used χ^2^ tests of difference to compare participant demographics with available demographic information about SCCM members who were sent the survey (data provided by SCCM). Free-text comments were manually reviewed by 2 independent reviewers (E.M. and K.K.) to identify themes. Disagreements were resolved by discussion and input from a third reviewer (H.C.P.) as needed.

Participant responses following randomized factors were assessed using multivariable logistic regression models. For cases 1 to 6, we performed separate multivariable logistic regression models to assess the association of randomized clinical factors with respondent recommendations for additional fluids and vasopressors, both overall and for each case pair. The factors randomized in the case pair were included as covariates in the regressions (eg, in cases 1 and 2, fluid volume received, MAP, volume examination, and medical history). For cases 7 to 10, an overall multivariable logistic regression model was performed to assess the association of randomized factors (vasopressor dose, dose trend, duration, and PIV location) with the recommendation to place a central line. Case number was also included as a covariate in all models to capture differences related to case stems. Details of the regression models are provided in the eMethods in Supplement 1. When more than 1 case was combined in a regression, a multilevel model was used to account for clustering by participant, with randomized factors treated as fixed effects and participant identification number treated as a random effect. Results are reported as an adjusted proportion of respondents with a 95% CI. These adjusted proportions of respondents represent average (mean) predicted probabilities, which were calculated using predictive margins after fitting each model. Odds ratios (ORs) are also provided in eTable 1 and eTable 2 in Supplement 1. Statistical significance was a 2-sided *P* < .05.

Data analysis was completed in StataMP version 17.0 (StataCorp) and Python version 3.10.9 (Python Software Foundation). Data analysis was conducted from February to September 2023.

## Results

The survey was electronically sent to 11 203 US-based SCCM member physicians and APPs, of whom 669 (6.0%) reviewed the study information page and 550 (4.9%) completed at least 1 vignette and were included. Of the 550 respondents (261 men [47.5%] and 192 women [34.9%]; 173 with >15 years of practice [31.5%]), 337 (61.3%) were physicians, 369 (67.1%) were critical care trained, and 294 (53.5%) were in academic practices. The remaining respondents were mostly APPs (101 respondents [18.4%]) and at practice locations other than an academic or private practice (91 respondents [16.6%]). Respondents had a range of experience levels and practice locations (Table 1). Compared with available demographic information about SCCM members who received the survey invitation, respondents had a similar gender distribution and practice region; however, there was a lower percentage of physicians (337 [71.7%] vs 8804 [78.6%]) among survey respondents (eTable 3 in Supplement 1). The majority of respondents found the cases to be realistic (251 of 477 [52.6%] found cases very realistic and 204 of 477 [42.8%] found cases somewhat realistic) (eFigure 1 in Supplement 1).

**Table 1.** Participant Characteristics and Practice Settings

Characteristics	Respondents, No (%) (N = 550)^a^
Gender	
Man	261 (47.5)
Woman	192 (34.9)
Other, preferred not to answer, or missing^b^	97 (17.6)
Years in practice	
In training	51 (9.3)
1-5	114 (20.7)
6-15	127 (23.1)
>15	173 (31.5)
Clinical role	
Physician	337 (61.3)
Advanced practice clinician	101 (18.4)
Other	32 (5.8)
Clinical practice type	
Academic	294 (53.5)
Private	83 (15.1)
Other	91 (16.6)
Trained in critical care	
Yes	369 (67.1)
No	100 (18.2)
ICU type	
Medical	118 (21.5)
Surgical	97 (17.6)
Mixed	221 (40.2)
Other	30 (5.5)
Beds in ICU, No.	
0-20	205 (37.2)
21-40	206 (37.4)
≥41	58 (10.6)
Region of practice	
Northeast	141 (25.6)
Midwest	121 (22.0)
South	118 (22.0)
West	88 (16.9)
Outside the US	2 (0.4)

Abbreviation: ICU, intensive care unit.

^a^ A total of 550 respondents completed at least 1 clinical vignette and were included in the primary analysis. Demographic questions were asked at the end of the survey. Missingness ranged from 51 respondents (10.0%) for region of practice to 85 respondents (15.5%) for years in practice.

^b^ Other included nonbinary or a third gender.

In questions about usual practice, most respondents self-reported targeting MAP of 65 mm Hg or greater (408 respondents [86.4%]) and administering 2 to 3 L of initial fluid (360 respondents [76.4%]) (eFigure 2 in Supplement 1). Respondents were divided between starting vasopressors during (225 respondents [47.5%]) or after (246 respondents [51.9%]) initial fluid resuscitation. A minority (179 respondents [38.1%]) reported often or always placing a central line to start vasopressors.

### Fluids and Vasopressors Decisions

Of the 550 respondents, 498 (90.5%) completed all cases 1 through 6. Responses varied across cases. Only 9 of the 498 respondents (1.8%) did not give fluids in any case and 22 (4.4%) gave fluids in all 6 cases. Similarly, 3 respondents (0.6%) did not start vasopressors in any case, while 134 (26.9%) started vasopressors in all 6 cases (eFigure 3 in Supplement 1). There was less response variation in cases where respondents saw the same fluid volume received; in cases with the same fluid volume received, 104 of 323 respondents (32.2%) to 154 of 328 (47.0%) of respondents’ recommendations for fluid depended on the case and 58 of 323 respondents (18.0%) to 161 of 346 (46.4%) of respondents’ recommendations for vasopressors depended on the case (eFigure 4 in Supplement 1). Reported Likert scale–based case difficulty was similar across cases and was not associated with fluid volume received or MAP. The adjusted proportion of respondents rating cases as difficult (ie, very difficult or somewhat difficult) ranged from 7.0% (95% CI, 4.8%-9.3%) to 13.1% (95% CI, 10.1%-16.1%) (eTable 4 in Supplement 1).

Fluid volume already received had the largest association with decisions about prescribing additional fluids and starting vasopressors. The overall adjusted proportion of respondents who prescribed additional fluid was 82.5% (95% CI, 80.2%-84.8%) after 1 L, 60.8% (95% CI, 57.7%-63.9%) after 2 L, and 17.5% (95% CI, 15.1%-19.9%) after 5 L (*P* < .001) (eTable 3 and eFigure 5 in Supplement 1). Conversely, the adjusted proportion of respondents who initiated vasopressors was 55.0% (95% CI, 51.9%-58.1%) after 1 L, 78.1% (95% CI, 75.5%-80.7%) after 2 L, and 92.7% (95% CI, 91.1%-94.3%) after 5 L (*P* < .001).

**Figure 1.**
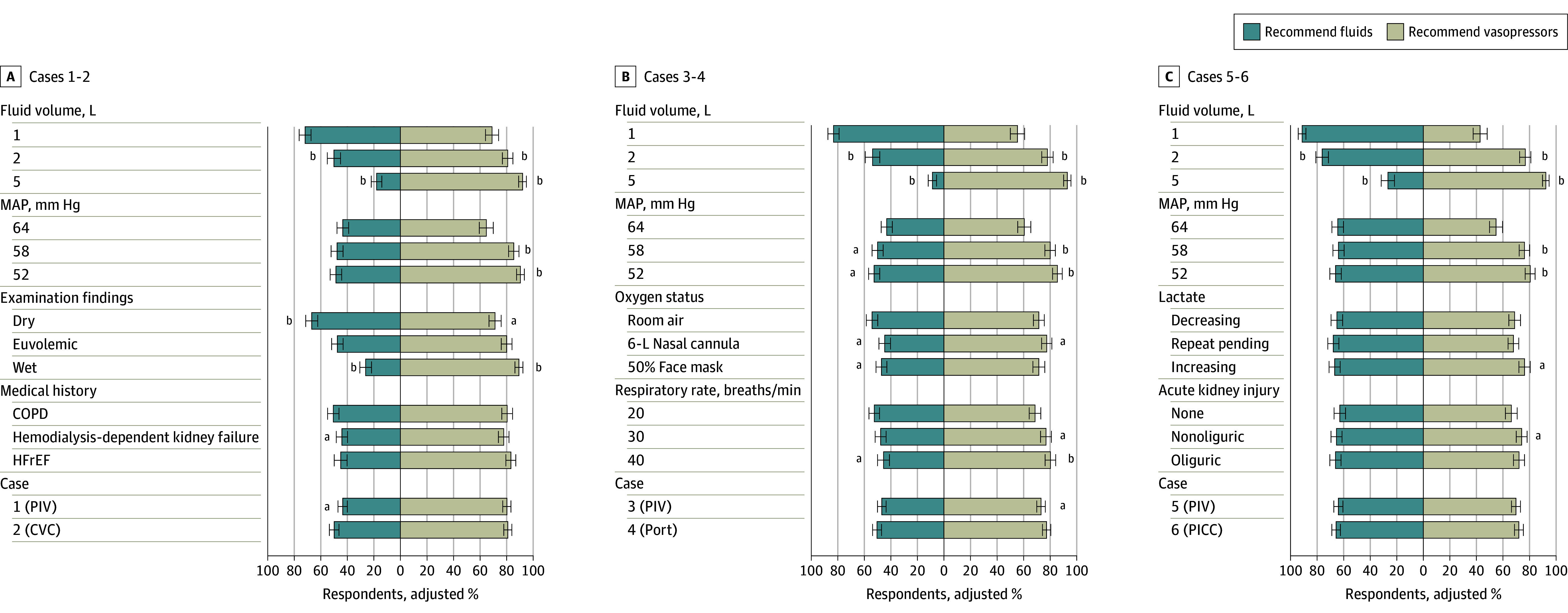
Adjusted Proportion of Respondents Recommending Fluids and Vasopressors From Logistic Regressions for Cases 1 to 6 The figure shows respondents recommendations for cases 1 and 2 (A), cases 3 and 4 (B), and cases 5 and 6 (C). Error bars indicate 95% CIs. In panel A, examination findings include dry (ie, dry mucus membranes), euvolemic (ie, moist mucous membranes and normal jugular venous pressure), and wet (elevated jugular venous pressure and bilateral pitting edema [grade +1]). In panel C, lactate findings include lactate decreasing (ie, initial lactate level of 36.94 mg/dL decreased to 24.32 mg/dL with fluids [to convert to millimoles per liter, multiply by 0.111]), lactate repeat pending (ie, initial lactate level of 36.94 mg/dL with repeat pending), and lactate increasing (ie, initial lactate level of 36.94 mg/dL increased to 48.65 mg/dL despite fluids). COPD indicates chronic obstructive pulmonary disease; CVC, central venous catheter; HFrEF, heart failure with reduced ejection fraction; MAP, mean arterial pressure; PICC, peripherally inserted central catheter; and PIV, peripheral venous catheter. ^a^Indicates *P* < .05. ^b^Indicates *P* < .001.

Clinical assessment of a patient’s volume status also had significant associations with fluid and vasopressor decisions. When a patient was assessed as having dry volume status, an adjusted 66.9% (95% CI, 62.5%-71.2%) of respondents prescribed additional fluid and an adjusted 70.8% (95% CI, 66.2%–75.3%) initiated vasopressors. Conversely, when the patient was assessed as having wet volume status (ie, overload), an adjusted 26.5% (95% CI, 22.3%-30.6%) of respondents prescribed additional fluid while an adjusted 89.3% (95% CI, 86.3%-92.2%) initiated vasopressors (Figure 1 and eTable 4 in Supplement 1).

Several other factors had statistically significant but small associations with the recommendation for additional fluids including MAP, history of kidney failure and receiving dialysis, oxygen requirement, and respiratory rate. History of HFrEF, lactate trend, and AKI were not associated with changes fluid decisions (Figure 1 and eTable 4 in Supplement 1).

MAP was associated with the decision to initiate vasopressors. The overall adjusted proportion of respondents initiating vasopressors when MAP levels were 52 mm Hg (85.2%; 95% CI, 83.1%-87.3%) or 58 mm Hg (80.9%; 95% CI, 78.6%-83.3%) were similar. In contrast, when MAP levels were 64 mm Hg, the adjusted proportion of respondents initiating vasopressors was only 59.1% (95% CI, 56.2%-62.1%) (*P* < .001) (eTable 3 and eFigure 5 in Supplement 1). Oxygen requirement, higher respiratory rate, rising lactate with fluids, and nonoliguric AKI all had significant but small associations with vasopressor initiation (favoring vasopressor use). History of kidney failure and receiving dialysis, HFrEF, downtrending lactate with fluids, and oliguric AKI were not associated with vasopressor initiation. Case number had a significant association with recommendation for fluids in case 1 vs 2 (adjusted OR, 0.63; 95% CI, 0.45-0.88; *P* = .007) and vasopressors in case 3 vs 4 (adjusted OR, 0.66; 95%CI, 0.45-0.97; *P* = .04) (Figure 1 and eTable 4 in Supplement 1).

For cases 1 through 6, 189 respondents requested additional information using free-text comment boxes which were manually reviewed. The most commonly requested information was patient weight (requested by 48 respondents [8.7%]) and point-of-care ultrasonography (requested by 39 respondents [7.1%]) (eTable 5 in Supplement 1).

### Route for Vasopressor Initiation

**Figure 2.**
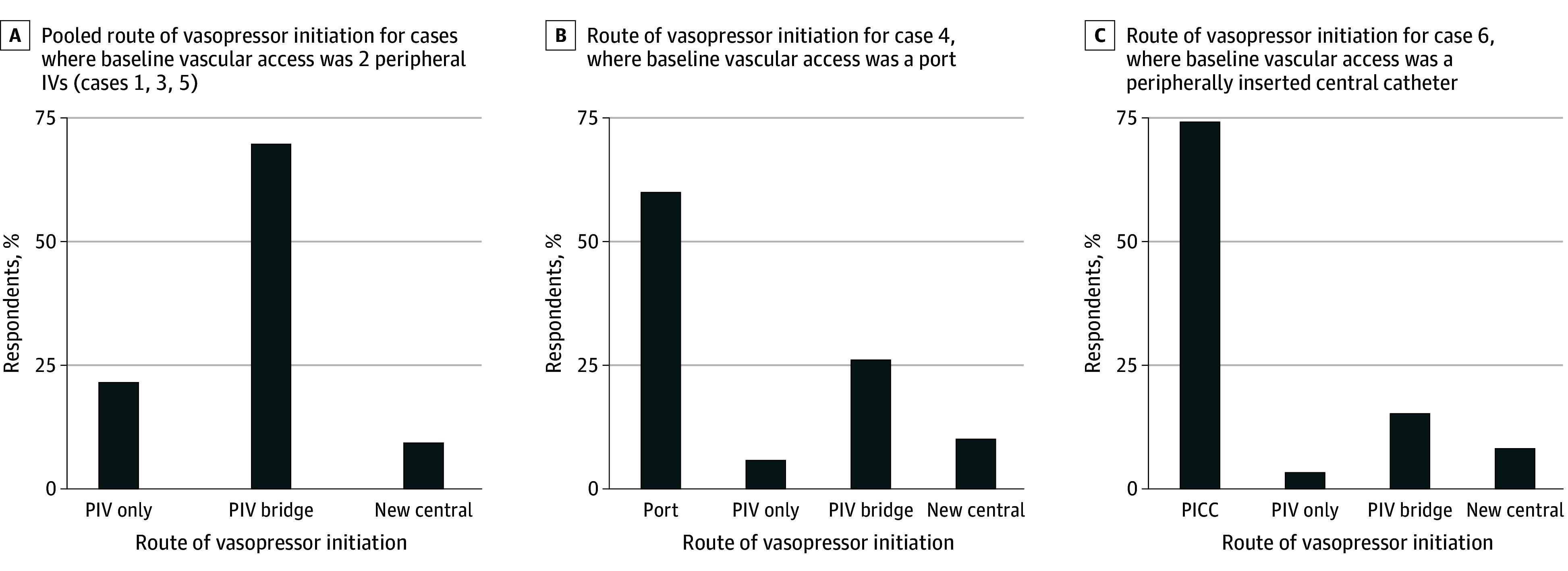
Preferred Route of Vasopressor Initiation Among Respondents Who Chose to Start Vasopressors The figure shows the pooled route of vasopressor initiation for cases 1, 3, and 5, where baseline vascular access was 2 PIVs (A; based on 1127 vignettes), the route of vasopressor initiation for case 4, where baseline vascular access was a port (B; based on 390 vignettes), and the route of vasopressor initiation for case 6, where baseline vascular access was a PICC (C; based on 367 vignettes). Case 2 was not included in this analysis because the patient in case 2 had a preexisting new temporary central line, which was presumed to be the default route of vasopressor initiation. PICC indicates peripherally inserted central catheter; PIV, peripheral venous catheter. PIV only refers to starting the vasopressor peripherally; PIV bridge refers to starting the vasopressor peripherally but planning to place a new central line; and new central refers to placing a new central line before starting the vasopressor.

In most vignettes (1023 of 1127 [90.8%]) where the patient’s baseline vascular access was PIVs, respondents chose peripheral vasopressor initiation, primarily with a plan to bridge to central access (782 respondents [69.4%]) (Figure 2A). No patient factors were associated with the route of vasopressor initiation in these cases (eTable 6 in Supplement 1). When a preexisting PICC or port was present in the vignette, a majority of respondents chose this route for vasopressor initiation (PICC, 272 respondents [74.1%] vs port, 233 respondents [59.7%]) (Figure 2B and Figure 2C).

### Route for Ongoing Vasopressor Use

**Figure 3.**
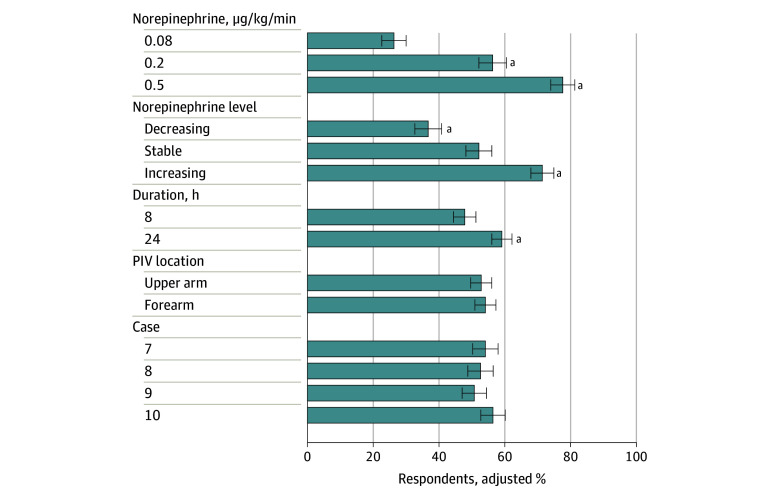
Adjusted Proportion of Respondents Recommending Placing Central Access From Logistic Regression for Cases 7 to 10 Patients in these cases were receiving norepinephrine through an 18-gauge peripheral venous catheter (PIV; location excluded the antecubital fossa). Respondents were asked if they would continue the vasopressor peripherally or place central access (ie, a temporary central line or peripherally inserted central catheter). Error bars denote 95% CIs. Case 7 was a 55-year-old female patient with cholecystitis; case 8 was a 70-year-old male patient with pneumonia; case 9 was a 65-year-old female patient with urosepsis; and case 10 was a 60-year-old male with cellulitis. ^a^Indicates *P* < .001.

Of the 550 respondents, 478 (86.9%) respondents completed all cases 7 through 10. Responses varied across cases with only 44 of the 478 respondents (9.2%) never placing a central line and 70 (14.6%) always placing a central line (eFigure 3 in Supplement 1). The adjusted proportion of respondents recommending a central line increased from 25.2% (95% CI, 21.8%-28.5%) for a lower dose of norepinephrine (0.08 µg/kg/min) to 78.0% (95% CI, 74.7%-81.2%) for a higher dose (0.50 µg/kg/min) (*P* < .001) (Figure 3 and eTable 7 in Supplement 1). Vasopressor dose trend was also associated with the decision to place central access; an adjusted 36.3% (95% CI, 32.8%-39.9%) of respondents recommended a central line with decreasing dose vs an adjusted 71.0% (95% CI, 67.7%-74.2%) with increasing dose (*P* < .001). Duration of vasopressor infusion had a significant but smaller association; the adjusted proportion of respondents recommending a central line was 47.1% (95% CI, 44.0%-50.1%) at 8 hours vs an adjusted 59.5% (95% CI, 56.6%-62.5%) at 24 hours (*P* < .001). Location of the PIV in the forearm vs upper arm and case number were not significantly associated with a decision for central line placement. In free-text comments, "In free-text comments, 33 respondents commented on additional information they would use to make decisions about central line placement, including anticipated procedures, hospital resources, policy, and other indications for central access (eTable 8 in Supplement 1). The most important self-reported factor informing the use of peripheral vasopressors was personal practice (242 of 476 respondents [50.8%]), followed by hospital policy (136 of 476 respondents [28.6%]) (eFigure 6 and eTable 9 in Supplement 1).

## Discussion

In this randomized vignette survey study of US critical care clinicians, we found that fluid volume already received was associated with the largest changes in decisions to administer additional fluids or initiate vasopressors in patients with sepsis. In contrast, other clinical factors (past medical history, respiratory status, lactate trend, and AKI) had small associations with these resuscitation decisions. Most respondents chose to start vasopressors peripherally, with higher or increasing vasopressor dose and longer duration associated with the subsequent decision to place a central line.

Personalization has been proposed as the future of sepsis resuscitation. The recent CLOVERS^17^ and CLASSIC^16^ trials found that fluid-liberal vs fluid-restrictive, vasopressor-early approaches result in similar patient outcomes. However, these trials have been criticized for their generalized approach to resuscitation, leading experts to call for personalization.^22,23^ At this time, there is no standard, accepted strategy for personalization. Many have suggested that clinical factors may help guide resuscitation. However, our study found that fluid volume received tended to outweigh other factors that would traditionally be considered for personalization, including volume status.

In our study, fluid volumes that respondents were willing to administer fell somewhere between 2 L to 5 L. Respondents self-reported administering 2 to 3 L in their usual practice. This finding aligned with case responses, where a majority of respondents prescribed fluid after 1 L, one-half prescribed fluid after 2 L, and few respondents prescribed fluid after 5 L, a volume associated with harm in retrospective studies and the maximal volume allowed in the fluid-liberal group of CLOVERS.^7,17^ The fluid volumes respondents were willing to administer align with the Surviving Sepsis Campaign (SSC) guidelines, which suggest an initial fluid volume of 30 mL/kg, or 2 to 3 L in an average adult.^24^ While we did not assess participant reasoning, in free-text responses, many respondents asked for patient weight, suggesting clinicians may be specifically using the 30 mL/kg guideline to make decisions about fluids or are using weight to personalize resuscitation.

Our results suggest that these fluid volumes are ingrained in practice and guide resuscitation decisions, while other clinical factors appear to play a much smaller role in personalizing resuscitation decisions, at least on average. For example, history of heart failure and kidney failure have historically been associated with increased risk for fluid overload.^25^ However, in our study, history of kidney failure and receiving dialysis had a small association with fluid administration decisions while history of HFrEF had no association. This finding may reflect the fact that clinicians are willing to give fluids to patients with these comorbidities, which is potentially beneficial, given evidence that suggests fluids may decrease mortality in these patients.^26^ In contrast, respiratory failure has been associated with harm from fluid resuscitation in sepsis trials in lower-resource settings.^27,28,29^ Additionally, in the CLASSIC trial,^16^ there was a signal for potential harm among patients on respiratory support in the fluid-liberal group. Yet, in our survey, oxygen requirement and respiratory rate only had small associations with resuscitation decisions, outweighed by fluid volume. Finally, there is some evidence to support guiding fluid resuscitation by lactate clearance, as suggested in the SSC guidelines.^24,30,31^ Despite this guideline, we found that lactate trend was not associated with changes in clinical decision-making.

These findings may suggest that apart from fluid volume received, clinicians are not using clinical factors to personalize resuscitation decisions. However, there are several potential alternative explanations for this apparent lack of personalization (Table 2). Importantly, our study only provides an average measure of personalization, measuring effects in the same direction. However, clinicians may be using clinical factors to personalize care in more nuanced ways. Indeed, we observed a range of responses across cases, even in cases where respondents saw the same fluid volume received (eg, all 1 L), which suggests potential unmeasured personalization was occurring.

**Table 2.** Limitations and Possible Explanations for the Finding of Lack of Personalization

Possible explanation	Details
True effect	Clinicians were not personalizing resuscitation in practice.
Average effect	Clinicians were personalizing resuscitation, but the key factors underlying personalization were exerting effects in opposite directions (eg, leading some clinicians to give more fluids and others to give less), resulting in a neutral average effect.
Wrong method	Vignettes were not able to measure personalization.
Wrong respondents	The clinicians we surveyed were not the ones making decisions about personalization in practice (eg, intensive care unit attending physicians were making decisions rather than trainees or internal medicine clinicians); however, this explanation is considered less likely given survey respondents represented clinicians across a range of positions and levels of training.
Wrong factors	Resuscitation was being personalized using other factors, such as dynamic measures of fluid responsiveness.

There are also other tools besides clinical factors that can be used to personalize resuscitation. For example, dynamic measures of fluid-responsiveness may help determine when patients need more fluids vs vasopressors. While these tools have been validated to assess fluid-responsiveness, their role in improving patient outcomes in sepsis has not been well studied.^32^ Yet, in free-text responses, many respondents requested dynamic fluid-responsiveness measures (eg, point-of-care ultrasonography or noninvasive cardiac output monitoring), suggesting that clinicians are using these bedside tools. More work is needed to understand how dynamic tools are used in practice and to determine their association with patient outcomes.

Our results also provide valuable information about peripheral vasopressor practices. We found that peripheral vasopressor initiation was commonly tolerated, with a vast majority of respondents willing to start vasopressors through a PIV if this was the patient’s only access. A number of respondents also chose to start vasopressors through a PIV over preexisting central access, perhaps due to concerns about line infections, although more work is needed to understand this observed practice. Decisions about peripheral vasopressor initiation were not associated with clinical factors, even hypotension severity, suggesting the decision about route of initiation was made independently from the decision to initiate vasopressors and may have been guided by other factors, such as personal practice or hospital policy. Beyond initiation, approximately one-half of respondents appeared willing to continue peripheral vasopressors in at least some patients, basing decisions about when to place a central line on vasopressor dose, trend, and duration, factors that are included in many hospital policies.^33^ This finding suggests that many clinicians are comfortable using peripheral vasopressors at low doses for durations up to 24 hours, which aligns with available safety data.^34,35^

### Limitations

This study has several limitations. First, it was a survey study and, thus, did not directly measure clinician practices. However, we used an evidence-based vignette survey method that has been shown to elicit preferences and predict behavior, allowing us to approximate clinical decision-making.^18,19,20,36^ Furthermore, the majority of respondents found cases to be realistic. Second, while many clinical factors were randomized, fixed factors (eg, age) were included in cases to enhance their realism. However, for all but 2 case pairs where there was a small association, case was not associated with participant answers, suggesting these fixed factors were not associated with clinician decisions. Conversely, it is possible that factors that were not included in the vignettes (eg, dynamic measures of fluid-responsiveness) may have been important for guiding resuscitation decisions and, therefore, our results may only reflect how clinicians would practice in situations where they did not have access to their preferred bedside fluid assessment tools. We allowed free-text responses to help capture such other important factors. Additionally, survey response bias could limit generalizability. However, our cohort was characteristically similar to the SCCM members who received the survey, suggesting our findings may be at least generalizable to the community of critical care clinicians within SCCM. Further, randomization of case order helped protect against selection bias introduced by variation in survey response.

## Conclusions

The findings of this survey study provide important insight into clinician decision-making in early sepsis resuscitation. Fluid volume already received was the predominant factor associated with decisions about additional fluids and vasopressors, outweighing many other clinical factors. Peripheral vasopressor use was common. Future trials aimed at personalizing resuscitation should account for the association of received fluid volume and should work to validate easily accessible bedside tools beyond clinical factors, such as dynamic fluid-responsiveness measures, to help guide personalization.

## Notes

Supplement 1. **eAppendix 1.** Outline of Randomized Clinical Factors **eAppendix 2.** Full Survey **eMethods.** Details of Logistic Regression Models **eTable 1.** Association of Fluid Volume and MAP With Recommendations for Fluids and Vasopressors, Cases 1-6 **eTable 2.** Association of Randomized Clinical Factors With Recommendations for Fluids and Vasopressors **eTable 3.** Characteristics of Survey Participants Compared to Characteristics of Society of Critical Care Medicine (SCCM) Providers Who Received the Survey **eFigure 1.** Perceived Realism of Clinical Vignettes **eFigure 2.** Self-Reported Usual Practices for Managing Patients With New Sepsis-Induced Hypotension **eFigure 3.** Range of Respondent Answers Across Cases **eFigure 4.** Range of Respondent Answers Across Cases Where Participants Saw the Same Fluid Volume Already Received **eTable 4.** Association of Fluid Volume, MAP, and Case With Perceived Case Difficulty, Cases 1-6 **eFigure 5.** Overall Association of Fluid Volume and MAP With Recommendations for Fluids and Vasopressors, Cases 1-6 **eTable 5.** Additional Requested Factors for Fluid and Vasopressor Decisions, Cases 1-6 **eTable 6.** Association of Randomized Clinical Factors With Peripheral Vasopressor Initiation **eTable 7.** Association of Randomized Clinical Factors With Recommendation to Place a Central Line in Patients Receiving Peripheral Vasopressors **eTable 8.** Identified Themes From a Free-Text Question Eliciting Factors That Contributed to Respondents’ Decision to Place a Central Line in Patients on Peripheral Vasopressors, Cases 7-10 **eFigure 6.** Self-Reported Factor That Most Influences the Decision to Use Peripheral Vasopressors **eTable 9.** Identified Themes From a Free-Text Question About Most Important Factors Impacting Decision About Peripheral Vasopressor Use

Supplement 2.
Data Sharing Statement
