# Supplementary material for: Understanding How Clinicians Personalize Fluid and Vasopressor Decisions in Early Sepsis Management
Source: JAMA Netw Open. 2024 Apr 19;7(4):e247480. doi: 10.1001/jamanetworkopen.2024.7480 (PMC11031682; doi:10.1001/jamanetworkopen.2024.7480)
Supplement: Supplement 1. — eAppendix 1. Outline of Randomized Clinical Factors eAppendix 2. Full Survey eMethods. Details of Logistic Regression Models eTable 1. Association of Fluid Volume and MAP With Recommendations for Fluids and Vasopressors, Cases 1-6 eTable 2. Association of Randomized Clinical Factors With Recommendations for Fluids and Vasopressors eTable 3. Characteristics of Survey Participants Compared to Characteristics of Society of Critical Care Medicine (SCCM) Providers Who Received the Survey eFigure 1. Perceived Realism of Clinical Vignettes eFigure 2. Self-Reported Usual Practices for Managing Patients With New Sepsis-Induced Hypotension eFigure 3. Range of Respondent Answers Across Cases eFigure 4. Range of Respondent Answers Across Cases Where Participants Saw the Same Fluid Volume Already Received eTable 4. Association of Fluid Volume, MAP, and Case With Perceived Case Difficulty, Cases 1-6 eFigure 5. Overall Association of Fluid Volume and MAP With Recommendations for Fluids and Vasopressors, Cases 1-6 eTable 5. Additional Requested Factors for Fluid and Vasopressor Decisions, Cases 1-6 eTable 6. Association of Randomized Clinical Factors With Peripheral Vasopressor Initiation eTable 7. Association of Randomized Clinical Factors With Recommendation to Place a Central Line in Patients Receiving Peripheral Vasopressors eTable 8. Identified Themes From a Free-Text Question Eliciting Factors That Contributed to Respondents’ Decision to Place a Central Line in Patients on Peripheral Vasopressors, Cases 7-10 eFigure 6. Self-Reported Factor That Most Influences the Decision to Use Peripheral Vasopressors eTable 9. Identified Themes From a Free-Text Question About Most Important Factors Impacting Decision About Peripheral Vasopressor Use [file jamanetwopen-e247480-s001.pdf]

Supplemental Online Content

Munroe ES, Weinstein J, Gershengorn HB, et al. Understanding how clinicians personalize fluid and vasopressor decisions in early sepsis management. JAMA Netw Open. 2024;7(4):e247480. doi:10.1001/jamanetworkopen.2024.7480

eAppendix 1. Outline of Randomized Clinical Factors
eAppendix 2. Full Survey
eMethods. Details of Logistic Regression Models
eTable 1. Association of Fluid Volume and MAP With Recommendations for Fluids and Vasopressors, Cases 1-6
eTable 2. Association of Randomized Clinical Factors With Recommendations for Fluids and Vasopressors
eTable 3. Characteristics of Survey Participants Compared to Characteristics of Society of Critical Care Medicine (SCCM) Providers Who Received the Survey
eFigure 1. Perceived Realism of Clinical Vignettes
eFigure 2. Self-Reported Usual Practices for Managing Patients With New Sepsis-Induced Hypotension
eFigure 3. Range of Respondent Answers Across Cases
eFigure 4. Range of Respondent Answers Across Cases Where Participants Saw the Same Fluid Volume Already Received
eTable 4. Association of Fluid Volume, MAP, and Case With Perceived Case Difficulty, Cases 1-6
eFigure 5. Overall Association of Fluid Volume and MAP With Recommendations for Fluids and Vasopressors, Cases 1-6
eTable 5. Additional Requested Factors for Fluid and Vasopressor Decisions, Cases 1-6.
eTable 6. Association of Randomized Clinical Factors With Peripheral Vasopressor Initiation
eTable 7. Association of Randomized Clinical Factors With Recommendation to Place a Central Line in Patients Receiving Peripheral Vasopressors
eTable 8. Identified Themes from a Free-Text Question Eliciting Factors That Contributed to Respondents' Decision to Place a Central Line in Patients on Peripheral Vasopressors, Cases 7-10
eFigure 6. Self-Reported Factor That Most Influences the Decision to Use Peripheral Vasopressors
eTable 9. Identified Themes From a Free-Text Question About Most Important Factors Impacting Decision About Peripheral Vasopressor Use

This supplemental material has been provided by the authors to give readers additional information about their work.

© 2024 Munroe ES et al. *JAMA Network Open.*

eAppendix 1. Outline of Randomized Clinical Factors

Factors randomized in Cases 1-6 (Fluid and vasopressor decisions)			
**Factor**	**Factor Levels**⊥	**Factor level details***	**Case**
Mean Arterial Pressure (MAP)	3	• 68/44 (MAP 52 mmHg)<br>• 74/48 (MAP 58 mmHg)<br>• 82/56 (MAP 64 mmHg)	1-6
Fluid volume	3	• 1L<br>• 2L<br>• 5L	1-6
Past Medical History (e.g., risk factors for fluid overload)	3	• COPD<br>• and Heart Failure with Reduced Ejection Fracture<br>• and End-Stage Renal Disease on dialysis	Case 1&2
Volume exam	3	• Moist mucus membranes and normal jugular venous pressure<br>• Dry mucus membranes<br>• Elevated jugular venous pressure and 1+ pitting bilateral lower extremity edema	Case 1&2
Oxygen support	3	• Room air<br>• 6 Liters Nasal Cannula<br>• 50% Face Mask	Case 3&4
Respiratory rate (RR)	3	• RR 20, no accessory muscle use<br>• RR 30, with mild accessory muscle use<br>• RR 40, with notable accessory muscle use	Case 3&4
Lactate trend	3	• Lactate has risen from 4.1 mmol/L to 5.4 mmol/L despite fluids<br>• Lactate has decreased from 4.1 mmol/L to 2.7mmol/L with fluids<br>• Initial lactate was 4.1mmol/L, repeat pending	Case 5&6
AKI (acute kidney injury)	3	• AKI with minimal urine output since arrival<br>• AKI with 200 cc (50ml/hr) urine output since arrival<br>• Creatinine at baseline	Case 5&6

Legend: Clinical factors that were randomized in cases 1-6. Fluid volume and MAP appeared in all cases 1-6. All other factors appeared only in pairs of cases, as designated in the Case column.
⊥Factor levels indicate the number of possible factors that were randomized.
*Text that participants were randomly assigned to see within the cases. In the full survey (Appendix B), this is the text that would appear in the blue brackets. For example, {MAP} would be randomly assigned to populate with any of the listed MAPs in this column.

© 2024 Munroe ES et al. *JAMA Network Open.*

Factors randomized in cases 7-10 (central line placement decision)		
**Factor**	**Factor Levels**[⊥]	**Factor Levels Detail***
Dose	3	• Low (0.08 mcg/kg/min or 5 mcg/min)<br>• Medium (0.2 mcg/kg/min or 15 mcg/min)<br>• High (0.5 mcg/kg/min or 35 mcg/min)
Trend	3	• Rising<br>• Stable<br>• Falling
Duration	2	• 8 hours<br>• 24 hours
Location	2	• Forearm, above wrist<br>• Upper arm, above Antecubital Fossa
Legend: Clinical factors that were randomized in cases 7-10. All factors appeared in all cases. For all cases, the presented scenario was a patient with norepinephrine running through an 18 Gauge peripheral IV.<br>[⊥]Factor levels indicate the number of possible factors that were randomized.<br>*Text that participants were randomly assigned to see within the cases. In the full survey (Appendix B), this is the text that would appear in the blue brackets. For example, {dose} would be randomly assigned to populate with any of the listed doses in this column.		

eAppendix 2: Full Survey*
*Of note, randomized clinical factors are denoted in blue brackets {factor}. Clinical factors that were randomized for each question are listed in Appendix A, above.

Vasopressor Practices

Start of Block: Consent

Background and Consent:
This brief 8-10 minute survey is being conducted to understand when and how providers start vasopressors in patients with early sepsis.

The survey has 3 sections:
- Sections 1 & 2 include 10 total short clinical cases
- Section 3 includes questions about your general practices and clinical background

This study has been reviewed and approved by the Institutional Review Board at University of Michigan. To keep your information confidential, we will not ask for your name or identifiable information during the survey. Your answers will not be traceable back to you.

Participation is voluntary. Refusal to participate will involve no penalty or loss of benefits to which you are otherwise entitled. You may decline to participate, refuse to answer any questions you prefer not to answer, or discontinue participation at any time. There is no cost to participate in this survey.

As a thank you for participating in this survey, you will be invited to enter a drawing for one of twenty $50 Amazon gift cards. Instructions are provided at the end of the survey.

By clicking the "next" button, you agree to participate in this research study. If you do not wish to participate in the study, please exit the survey now.

Thank you for your help.

End of Block: Consent

Start of Block: Instructions

Q2 **Section 1: Cases 1-6**
You will be shown 6 short clinical cases and asked how you would manage these example patients who are presenting to your Intensive Care Unit with sepsis and hypotension. This

© 2024 Munroe ES et al. *JAMA Network Open.*

survey is meant to assess provider practices; it is not meant to be a test of knowledge.

Please choose the answer that most reflects what you would do in your clinical practice in each scenario based on the information provided.

End of Block: Instructions

Start of Block: Initial cases 1

X→

Q3 Mr. X is a 78 year old man with diabetes, coronary artery disease s/p RCA stent in 2012, and {Past Medical History} who was brought to the ED by his daughter after she found him confused at home. He was febrile and hypotensive on arrival. The ED team started broad spectrum antibiotics for suspected sepsis of unclear source.

On exam, patient is A&Ox1 with {volume exam}. SpO2 94% on room air. Labs are pending.

IV fluids received: {fluid volume}
Current BP: {MAP}
Access: 2 peripheral IVs

What would you do next to manage this patient's low blood pressure?

- ○ Give additional IV fluids (1)
- ○ Give additional IV fluids and initiate vasopressors (2)
- ○ Initiate vasopressors without giving additional IV fluids (3)
- ○ No additional intervention; monitor and re-assess (4)

Display This Question:

If Q3 = 2

Or Q3 = 3

X→

© 2024 Munroe ES et al. *JAMA Network Open.*

Q4 How would you start vasopressors?

- ○ Peripheral IV (1)
- ○ Peripheral IV but plan to place a central line (2)
- ○ Central line (3)

X→

Q124 How difficult was it to decide on the next step in management in this case?

- ○ Very difficult (1)
- ○ Somewhat difficult (2)
- ○ Neutral (3)
- ○ Somewhat easy (4)
- ○ Very easy (5)

Q5 (OPTIONAL) Do you have any other comments on this case?

____

Page Break

© 2024 Munroe ES et al. *JAMA Network Open.*

End of Block: Initial cases 1

Start of Block: Initial cases 2

X→

Q6 Mr. W is a 74 year old man with diabetes, atrial fibrillation, and {Past Medical History} who presented to an outside hospital ED 3 hours ago with a left leg wound infection. The outside hospital has started appropriate antibiotics and is transferring the patient to your institution for surgical evaluation given concern for necrotizing fasciitis.

On exam, patient is A&Ox1. He has a left lower extremity wound with surrounding erythema, {volume exam}. SpO2 95% on room air. Labs are pending.

IV fluids received: {fluid volume}
Current BP: {MAP}
Access: Central line placed at the outside hospital due to difficult IV access.

What would you do next to manage this patient's low blood pressure?

- ○ Give additional IV fluids (1)
- ○ Give additional IV fluids and initiate vasopressors (2)
- ○ Initiate vasopressors without giving additional IV fluids (3)
- ○ No additional intervention; monitor and re-assess (4)

X→

© 2024 Munroe ES et al. *JAMA Network Open.*

Q125 How difficult was it to decide on the next step in management in this case?

- ○ Very difficult (1)
- ○ Somewhat difficult (2)
- ○ Neutral (3)
- ○ Somewhat easy (4)
- ○ Very easy (5)

Q7 (OPTIONAL) Do you have any other comments on this case?

____

Page Break

© 2024 Munroe ES et al. *JAMA Network Open.*

End of Block: Initial cases 2

Start of Block: Initial cases 3

X→

Q8 Mr. M is a 66 year old man with a history of non-small cell lung cancer s/p chemo/radiation therapy in 2020 who presents with 2 days of fever and cough. Chest x-ray shows a left lower lobe pneumonia. The ED team has started appropriate antibiotics.

On your evaluation, SpO2 is 94% on {oxygen support} and {respiratory rate}. Patient is A&Ox3, mucus membranes are moist, no lower extremity edema. Labs are pending.

IV fluids received: {fluid volume}
Current BP: {MAP}
Access: 2 peripheral IVs

What would you do next to manage this patient's low blood pressure?

○ Give additional IV fluids (1)

○ Give additional IV fluids and initiate vasopressors (2)

○ Initiate vasopressors without giving additional IV fluids (3)

○ No additional intervention; monitor and re-assess (4)

Display This Question:

If Q8 = 2

Or Q8 = 3

Q9 How would you start vasopressors?

○ Peripheral IV (1)

○ Peripheral IV but plan to place a central line (2)

○ Central line (3)

© 2024 Munroe ES et al. *JAMA Network Open.*

X→ X→

Q126 How difficult was it to decide on the next step in management in this case?

- Very difficult (1)
- Somewhat difficult (2)
- Neutral (3)
- Somewhat easy (4)
- Very easy (5)

Q10 (OPTIONAL) Do you have any other comments on this case?

____

Page Break

© 2024 Munroe ES et al. *JAMA Network Open.*

End of Block: Initial cases 3

Start of Block: Initial cases 4

Q11 Mr. N is a 68 year old man who was recently diagnosed with colon cancer. He presents to the ED with 2 days of fevers and cough. Chest x-ray is concerning for pneumonia. The ED team has started him on appropriate antibiotics.

On your evaluation, SpO2 is 93% on {oxygen support} and {respiratory rate}. Patient is A&Ox3, mucus membranes are moist, no lower extremity edema. Labs are pending.

IV fluids received: {fluid volume}
Current BP: {MAP}
Access: PORT in place for planned chemotherapy.

What would you do next to manage this patient's low blood pressure?

- ○ Give additional IV fluids (1)
- ○ Give additional IV fluids and initiate vasopressors (2)
- ○ Initiate vasopressors without giving additional IV fluids (3)
- ○ No additional intervention; monitor and re-assess (4)

Display This Question:

If Q11 = 2

Or Q11 = 3

X→ X→

© 2024 Munroe ES et al. *JAMA Network Open.*

Q12 How would you start vasopressors?

- ◯ Existing PORT (4)
- ◯ Peripheral IV (1)
- ◯ Peripheral IV but plan to place a new central line (2)
- ◯ New central line (3)

Q127 How difficult was it to decide on the next step in management in this case?

- ◯ Very difficult (1)
- ◯ Somewhat difficult (2)
- ◯ Neutral (3)
- ◯ Somewhat easy (4)
- ◯ Very easy (5)

Q13 (OPTIONAL) Do you have any other comments on this case?

____

Page Break

© 2024 Munroe ES et al. *JAMA Network Open.*

End of Block: Initial cases 4

Start of Block: Initial cases 5

X→

Q14 Ms. A is a 56 year old woman with obesity, obstructing renal stones s/p left nephrostomy tube, and recurrent urinary tract infections who presents to the ED with suspected urosepsis in the setting of 3 days of urinary frequency, left flank pain, and fevers. There is worsened left hydronephrosis on CT imaging. The ED team has started appropriate antibiotics based on prior culture data. They are also contacting Interventional Radiology and Urology about possible intervention.

On exam, patient is A&Ox3 and uncomfortable with left flank tenderness. Mucus membranes are moist, no lower extremity edema. SpO2 96% on room air.

Labs are notable for {lactate trend}. {AKI}

IV fluids received: {fluid volume}
Current BP: {MAP}
Access: 2 peripheral IVs

What would you do next to manage this patient's low blood pressure?

- ○ Give additional IV fluids (1)
- ○ Give additional IV fluids and initiate vasopressors (2)
- ○ Initiate vasopressors without giving additional IV fluids (3)
- ○ No additional intervention; monitor and re-assess (4)

Display This Question:

If Q14 = 2

Or Q14 = 3

X→

© 2024 Munroe ES et al. *JAMA Network Open.*

Q15 How would you start vasopressors?

- ○ Peripheral IV (1)
- ○ Peripheral IV but plan to place a central line (2)
- ○ Central line (3)

X→ X→

Q128 How difficult was it to decide on the next step in management in this case?

- ○ Very difficult (1)
- ○ Somewhat difficult (2)
- ○ Neutral (3)
- ○ Somewhat easy (4)
- ○ Very easy (5)

Q16 (OPTIONAL) Do you have any other comments on this case?

____

Page Break

© 2024 Munroe ES et al. *JAMA Network Open.*

End of Block: Initial cases 5

Start of Block: Initial cases 6

Q17 Ms. F is a 59 year old woman with obesity and a recent admission for acute cholecystitis s/p percutaneous cholecystostomy tube. She presents to the ED after dislodgement of the cholecystectomy tube with RUQ pain and fevers. The ED team is concerned about recurrent cholecystitis. They have started antibiotics and are consulting Interventional Radiology and Surgery for possible intervention.

On exam, patient is A&Ox3 with RUQ tenderness. Mucus membranes are moist, no lower extremity edema. SpO2 95% on room air.

Labs are notable for {lactate trend}. {AKI}

IV fluids received: {fluid volume}
Current BP: {MAP}
Access: Dual-lumen upper extremity PICC (peripherally inserted central catheter) still in place from patient's recent admission.

What would you do next to manage this patient's low blood pressure?

- ○ Give additional IV fluids (1)
- ○ Give additional IV fluids and initiate vasopressors (2)
- ○ Initiate vasopressors without giving additional IV fluids (3)
- ○ No additional intervention; monitor and re-assess (4)

Display This Question:

If Q17 = 2

Or Q17 = 3

© 2024 Munroe ES et al. *JAMA Network Open.*

Q18 How would you start vasopressors?

- ○ Existing PICC (4)
- ○ Peripheral IV (1)
- ○ Peripheral IV but plan to place a new central line (2)
- ○ New central line (3)

Q129 How difficult was it to decide on the next step in management in this case?

- ○ Very difficult (1)
- ○ Somewhat difficult (2)
- ○ Neutral (3)
- ○ Somewhat easy (4)
- ○ Very easy (5)

Q19 (OPTIONAL) Do you have any other comments on this case?

____

Page Break

© 2024 Munroe ES et al. *JAMA Network Open.*

End of Block: Initial cases 6

Start of Block: Peripheral VP instructions

Q20 **Section 2: Cases 7-10**
For cases 7-10, you are receiving hand-off about the following patients who were recently admitted to the Intensive Care Unit for septic shock requiring vasopressors.

These patients are **currently receiving norepinephrine through an 18 gauge peripheral IV**.

Assume each patient has been adequately resuscitated and has adequate IV access for lab draws and the other medications they are receiving, including antibiotics and fluids.

End of Block: Peripheral VP instructions

Start of Block: Peripheral VP case 7

X→ X→

Q21

**Patient History**	**Norepinephrine Dose**	**Time norepinephrine has been infusiong through IV**	**Vascular access**
55F with cholecystitis	{dose}, {trend} over the past few ours	{duration}	18 Gauge IV in the {location}

How would you infuse norepinephrine for this patient during your shift? (Choose one)

○ Continue using this peripheral IV (1)

○ Continue using this peripheral IV in the short term, but reassess need for a central line in the next few hours (2)

○ Place a central line for norepinephrine now (3)

○ Obtain alternative access for norepinephrine (other than a central line: e.g., a new peripheral IV, PICC) (4)

© 2024 Munroe ES et al. *JAMA Network Open.*

Display This Question:

If Q21 = 4

Q22 What alternative access would you place to administer norepinephrine?

- ◯ New peripheral IV (e.g., an IV that is larger, in a new location, or ultrasound-guided) (1)
- ◯ Midline catheter (2)
- ◯ PICC (peripherally-inserted central catheter) (3)
- ◯ Other (4) ____

Q23 (OPTIONAL) Do you have any other comments on this case?

____

End of Block: Peripheral VP case 7

Start of Block: Peripheral VP Case 8

© 2024 Munroe ES et al. *JAMA Network Open.*

Q24

Patient History	Norepinephrine Dose	Time norepinephrine has been infusiong through IV	Vascular access
70M with pneumonia	{dose}, {trend} over the past few ours	{duration}	18 Gauge IV in the {location}

How would you infuse norepinephrine for this patient during your shift? (Choose one)

- ◯ Continue using this peripheral IV (1)
- ◯ Continue using this peripheral IV in the short term, but reassess need for a central line in the next few hours (2)
- ◯ Place a central line for norepinephrine now (3)
- ◯ Obtain alternative access for norepinephrine (other than a central line: e.g., a new peripheral IV, PICC) (4)

Display This Question:

If Q24 = 4

X→

Q25 What alternative access would you place to infuse norepinephrine?

- ◯ New peripheral IV (e.g., an IV that is larger, in a new location, or ultrasound-guided) (1)
- ◯ Midline catheter (2)
- ◯ PICC (peripherally-inserted central catheter) (3)
- ◯ Other (4) ____

Q26 (OPTIONAL) Do you have any other comments on this case?

____

End of Block: Peripheral VP Case 8

© 2024 Munroe ES et al. *JAMA Network Open.*

Start of Block: Peripheral VP case 9

Q27

Patient History	Norepinephrine Dose	Time norepinephrine has been infusiong through IV	Vascular access
65F with urosepsis	{dose}, {trend} over the past few ours	{duration}	18 Gauge IV in the {location}

How would you infuse norepinephrine for this patient during your shift? (Choose one)

- ○ Continue using this peripheral IV (1)
- ○ Continue using this peripheral IV in the short term, but reassess need for a central line in the next few hours (2)
- ○ Place a central line for norepinephrine now (3)
- ○ Obtain alternative access for norepinephrine (other than a central line: e.g., a new peripheral IV, PICC) (4)

Display This Question:

If Q27 = 4

Q28 What alternative access would you place to infuse norepinephrine?

- ○ New peripheral IV (e.g., an IV that is larger, in a new location, or ultrasound-guided) (1)
- ○ Midline catheter (2)
- ○ PICC (peripherally-inserted central catheter) (3)
- ○ Other (4) ____

Q29 (OPTIONAL) Do you have any other comments on this case?

____

© 2024 Munroe ES et al. *JAMA Network Open.*

End of Block: Peripheral VP case 9

Start of Block: Peripheral VP case 10

Q30

Patient History	Norepinephrine Dose	Time norepinephrine has been infusiong through IV	Vascular access
60M with cellulitis	{dose}, {trend} over the past few ours	{duration}	18 Gauge IV in the {location}

How would you infuse norepinephrine for this patient during your shift? (Choose one)

○ Continue using this peripheral IV (1)

○ Continue using this peripheral IV in the short term, but reassess need for a central line in the next few hours (2)

○ Place a central line for norepinephrine now (3)

○ Obtain alternative access for norepinephrine (other than a central line: e.g., a new peripheral IV, PICC) (4)

Display This Question:

If Q30 = 4

Q31 What alternative access would you place to infuse norepinephrine?

○ New peripheral IV (e.g., an IV that is larger, in a new location, or ultrasound-guided) (1)

○ Midline catheter (2)

○ PICC (peripherally-inserted central catheter) (3)

○ Other (4) ____

© 2024 Munroe ES et al. *JAMA Network Open.*

Q32 (OPTIONAL) Do you have any other comments on this case?

____

End of Block: Peripheral VP case 10

Start of Block: Follow-up questions

Q33 How realistic were the cases presented in this survey?

- ○ Unrealistic (1)
- ○ Somewhat unrealistic (2)
- ○ Somewhat realistic (3)
- ○ Very realistic (4)

Q34 (OPTIONAL) Do you have any comments about the cases presented?

____

Q35 In your practice, for patients whose only indication for central access is vasopressor infusion, what factor most influences your decision to place a central line?

- ○ Hospital policy (1)
- ○ Nursing preference (2)
- ○ Personal practice (3)
- ○ Other (4) ____

End of Block: Follow-up questions

Start of Block: General Practice Pattern

© 2024 Munroe ES et al. *JAMA Network Open.*

Q36 **Section 3: General Practice**
Please answer the following 4 questions to best describe your general (average) practice for managing *new* patients presenting with sepsis and hypotension. We know that there are many factors that influence your management of individual patients. This section is meant to provide an overview of your general practice.

Q37 The amount of IV fluid I give within the first 6 hours of a patient's presentation is usually:

	0	1	2	3	4	5	6	7	8	9	10
Amount of fluid (liters) ()											

Q38 I target a MAP of:

	55	60	65	70	75	80	85
MAP (mmHg) ()							

Q39 If a patient's MAP is below my target, I usually start vasopressors

- ◯ Before giving fluid (1)
- ◯ While giving fluid (2)
- ◯ After giving fluid (3)
- ◯ Never, I don't use vasopressors (4)

© 2024 Munroe ES et al. *JAMA Network Open.*

Q40 I place a central line to start vasopressors:

- ○ Never (1)
- ○ Rarely (2)
- ○ Sometimes (3)
- ○ Often (4)
- ○ Always (5)

Q41 (OPTIONAL) Do you have any other comments about your usual practice?

____

End of Block: General Practice Pattern

Start of Block: Background

Q42 **Section 3: Background**
These final few questions ask for anonymous information about you and your clinical practice setting. You may choose to skip any questions that you are not comfortable answering.

X→

Q43 What is your clinical role?

- ○ Physician (1)
- ○ Advanced Practice Provider (NP or PA) (2)
- ○ Other (3) ____

© 2024 Munroe ES et al. *JAMA Network Open.*

Q45 To which gender identity do you most identify?

- Male (1)
- Female (2)
- Non-binary / third gender (3)
- Prefer not to answer (4)

Q46 How many years have you been in practice?

- Still in training (residency, fellowship) (1)
- 1-5 years (2)
- 6-10 years (3)
- 11-15 years (4)
- 15 or more years (5)

Q47 What percentage of your time do you spend providing direct patient care in an ICU setting?

- 0-25% (1)
- 25-50% (2)
- 50-75% (3)
- 75-100% (4)

© 2024 Munroe ES et al. *JAMA Network Open.*

Q44 What is your primary practice specialty? (choose all that apply)

- ☐ Critical Care (1)
- ☐ Internal Medicine (2)
- ☐ Surgery (3)
- ☐ Anesthesiology (4)
- ☐ Emergency Medicine (5)
- ☐ Other (6) ____

Q48 In what clinical environment do you primarily practice?

- ○ Private practice (1)
- ○ Academic (2)
- ○ Other (3) ____

Q49 In what type of ICU do you primarily practice?

- ○ Medical (1)
- ○ Surgical (2)
- ○ Mixed (3)
- ○ Other (4) ____

© 2024 Munroe ES et al. *JAMA Network Open.*

Q50 How many ICU beds are in the critical care unit where you primarily practice?

- ○ 0-10 beds (1)
- ○ 11-20 beds (2)
- ○ 21-30 beds (3)
- ○ 31-40 beds (4)
- ○ 41 or more beds (5)

Q51 Where do you primarily practice?

- ○ Northeastern US (1)
- ○ Midwestern US (2)
- ○ Southern US (3)
- ○ Western US (4)
- ○ Outside of the US (5)

End of Block: Background

Start of Block: Wrap-up

Q53 Follow this link to to enter your contact information if you would like to be entered into a drawing for a $50 Amazon Gift Card. Your name and email are collected separately to ensure that your responses to this survey remain anonymous.

Click the link above or copy this link into your browser:
https://umich.qualtrics.com/jfe/form/SV_exM1ii7thjuIDYy

Thank you for your time.

End of Block: Wrap-up

© 2024 Munroe ES et al. *JAMA Network Open.*

eMethods. Details of Logistic Regression Models

<u>Case 1-6</u>

A. **Fluids and vasopressor decisions**: We performed separate multivariable, multilevel logistic regression models to assess the association between the randomized clinical factors and respondent recommendations for 1) additional fluids and 2) vasopressors. We did this using both overall regressions (all cases 1-6) and regressions for each case pair, as outlined here:
    a. Overall regressions were performed pooling the results for cases 1-6. The goal of these regressions was to assess the overall association of fluid volume received and MAP with fluid/vasopressor recommendations. Fluid volume and MAP were randomized in all 6 cases. Factors were randomized individually for each case, so respondents could have theoretically seen the same fluid volume or MAP for all 6 cases. In these regressions, clinical factors from all cases were used as co-variates (fluid volume received, MAP, volume status exam, past medical history, oxygen requirement, respiratory rate, lactate trend, AKI). However, because clinical factors besides fluid volume and MAP were not randomized in all cases (e.g., oxygen requirement was randomized in cases 3 &4 but fixed as room air for cases 1, 2, 5, 6), the individual effects of these factors were not reported in the overall models. Case number was also included as a co-variate to capture the impact of differences between case stems. Results are presented in **e-Table 1 and e-Figure 4**.
    b. Separate regressions were then performed for each case pair (cases 1 and 2, cases 3 and 4, cases 5 and 6). The goal of these regressions was to assess the association of the randomized factors within each case pair with respondent recommendations in those cases. The factors randomized in the case pair were included as co-variates in each regression (e.g., in cases 1 and 2, co-variates were: fluid volume received, MAP, volume status exam, past medical history). Factors were randomized individually for each case, so respondents could have theoretically seen the same value, e.g., dry volume exam, for both cases in the pair. Case number was also included as a co-variate to capture the impact of differences between case stems. Results are presented in **Figure 2 and e-Table 2.**

B. **Peripheral vasopressor initiation**: Among respondents who recommended vasopressors, we performed multivariable logistic regression models to determine the association of randomized factors with the decision to start vasopressors peripherally (vs via central access). Given each case included a different combination of baseline access (i.e., PIV, pre-existing CVC, Port, or PICC) and randomized factors, separate regressions were performed for each case. Given regression models were run individually for each case and each respondent answered each case once, multilevel modeling was not used. We did not perform a regression for case 2 given this case presented a patient with a pre-existing, new CVC which is the traditional gold standard route for vasopressor administration. Results are presented in **e-Table 6.**

© 2024 Munroe ES et al. *JAMA Network Open.*

C. **Case difficulty**: We performed a multivariable, multilevel logistic regression model to assess the association between fluid volume received, MAP, and case number with reported case difficulty. A case was defined as difficult if the respondent answered “somewhat difficult” or “very difficult” on the 5-point Likert scale. Results are presented in **e-Table 4**.

<u>Cases 7-10</u>

For cases 7-10, an overall multivariable, multilevel logistic regression model was performed to assess the association between randomized factors and the recommendation to place a central line. All randomized factors were included as co-variates (vasopressor dose, dose trend, duration, and PIV location). Case number was also included as a co-variate to capture the impact of differences between case stems. Factors were randomized individually for each case, so respondents could have theoretically seen the same values for all 4 cases. Results are presented in **Figure 3 and e-Table 7.**

© 2024 Munroe ES et al. *JAMA Network Open.*

eTable 1. Association of Fluid Volume and MAP With Recommendations for Fluids and Vasopressors, Cases 1-6

	Recommend Fluids			Recommend Vasopressors		
	Odds Ratio (95% CI)	Adjusted Proportion of Respondents* Percent (95% CI)	P-value	Odds Ratio (95% CI)	Adjusted Proportion of Respondents* Percent (95% CI)	P-value
**Fluid Volume**						
1 Liter	Ref	82.5 (80.2, 84.8)		Ref	55.0 (51.9, 58.1)	
2 Liters	0.21 (0.16, 0.27)	60.8 (57.7, 63.9)	<0.001	5.09 (3.89, 6.67)	78.1 (75.5, 80.7)	<0.001
5 Liters	0.01 (0.01, 0.02)	17.5 (15.1, 19.9)	<0.001	29.10 (19.90, 42.54)	92.7 (91.1, 94.3)	<0.001
**MAP (blood pressure)**						
52 mmHg (68/44)	1.55 (1.20, 2.00)	56.2 (53.7, 58.8)	0.001	9.02 (6.59, 12.34)	85.2 (83.1, 87.3)	<0.001
58 mmHg (74/48)	1.30 (1.00, 1.67)	54.0 (51.4, 56.5)	0.046	5.71 (4.26, 7.65)	80.9 (78.6, 83.3)	<0.001
64 mmHg (82/56)	Ref	50.6 (48.0, 53.3)		Ref	59.1 (56.2, 62.1)	

Legend: Association of fluid volume and MAP with recommendations for fluids and vasopressors in cases 1-6. Odds ratios and adjusted proportion of respondents were determined using separate multivariable, multilevel logistic regression models for 1) the recommendation to prescribe fluid and 2) the recommendation to initiate vasopressors. Multilevel models were performed to allow clustering by participant ID, which was treated as a random effect. Fluid volume and MAP were randomized across all cases 1-6 and their overall effects are reported here. The other clinical factors included in cases 1-6 (volume status exam, medical history, oxygen requirement, respiratory rate, lactate trend, and acute kidney injury) were randomized in 2 out of 6 cases but kept constant in the other cases (e.g., oxygen requirement was randomized in cases 3 and 4 but kept constant at room air in cases 1, 2, 5, 6). These clinical factors were included as co-variates in these regressions, but given they were not randomized in each case, their overall effects are not reported. Effects of all randomized clinical factors are reported in separate case-paired regressions (in e-Table 2 and Figure 2). N=3,129 completed vignettes

*Adjusted proportion of respondents (also known as average predicted probabilities) were calculated using predictive margins after fitting each regression model. These predicted probabilities represent the proportion of respondents (as a percentage) recommending fluids or vasopressors based on the listed factors, after adjusting for all other factors in the model.

Definitions: CI= confidence interval, Ref= reference value, MAP= mean arterial pressure

© 2024 Munroe ES et al. *JAMA Network Open.*

© 2024 Munroe ES et al. *JAMA Network Open.*

eTable 2. Association of Randomized Clinical Factors WIth Recommendations for Fluids and Vasopressors

eTable 2a. Association of randomized clinical factors with recommendations for fluids and vasopressors, Cases 1 and 2†

	Recommend Fluids			Recommend Vasopressors		
	Odds Ratio (95% CI)	Adjusted Proportion of Respondents* Percent (95% CI)	P-value	Odds Ratio (95% CI)	Adjusted Proportion of Respondents* Percent (95% CI)	P-value
**Fluid Volume**						
1 Liter	Ref	72.2 (67.9, 76.6)		Ref	68.2 (63.6, 72.8)	
2 Liters	0.25 (0.15, 0.39)	50.0 (45.1, 54.8)	<0.001	2.82 (1.66, 4.79)	80.8 (76.8, 84.8)	<0.001
5 Liters	0.03 (0.01, 0.06)	18.0 (14.2, 21.8)	<0.001	12.40 (5.88, 26.14)	92.3 (89.6, 95.0)	<0.001
**MAP (blood pressure)**						
52 mmHg (68/44)	1.45 (0.94, 2.24)	48.7 (44.4, 53.1)	0.094	10.67 (5.53, 20.60)	89.8 (86.8, 92.8)	<0.001
58 mmHg (74/48)	1.34 (0.87, 2.07)	47.6 (43.2, 52.0)	0.186	6.41 (3.42, 12.0)	85.9 (82.4, 89.5)	<0.001
64 mmHg (82/56)	Ref	43.5 (39.0, 47.9)		Ref	64.4 (59.5, 69.2)	
**Volume status**						
Dry	3.77 (2.35, 6.05)	66.9 (62.5, 71.2)	<0.001	0.44 (0.26, 0.76)	70.8 (66.2, 75.3)	0.003
Wet	0.24 (0.15, 0.38)	26.5 (22.3, 30.6)	<0.001	3.02 (1.67, 5.45)	89.3 (86.3, 92.2)	<0.001
Euvolemic	Ref	47.2 (42.6, 51.9)		Ref	80.0 (76.1, 83.9)	
**Medical History**						
COPD	Ref	50.9 (46.5, 55.2)		Ref	80.0 (76.1, 83.9)	
ESRD on HD	0.63 (0.41, 0.97)	44.2 (39.9, 48.6)	0.034	0.80 (0.47, 1.36)	77.7 (73.8, 81.7)	0.415
HFrEF	0.65 (0.42, 1.01)	44.8 (40.4, 49.2)	0.054	1.43 (0.82, 2.48)	83.3 (779.8, 86.8)	0.208
**Case**						
Case 1 (PIV)	0.63 (0.45, 0.88)	43.4 (40.0, 46.9)	0.007	0.96 (0.64, 1.43)	80.2 (77.2, 83.3)	0.842
Case 2 (CVC)	Ref	49.9 (46.4, 53.4)			80.6 (77.6, 83.7)	

e-Table 4a Legend: Association of randomized clinical factors with participant recommendation for fluids and vasopressors in cases 1 and 2. Odds ratios and adjusted proportion of respondents were determined using separate multivariable, multilevel logistic regression models for 1) the recommendation to prescribe fluid and 2) the recommendation to initiate vasopressors. Multilevel models were performed to allow clustering by participant ID, which was treated as a random effect. N=1,043 completed vignettes

†Corresponds to Figure 1a.

*Adjusted proportion of respondents (also known as average predicted probabilities) were calculated using predictive margins after fitting each regression model. These predicted probabilities represent the proportion of respondents (as a percentage) recommending fluids or vasopressors based on the listed factors, after adjusting for all other factors in the model.

Definitions: CI= confidence interval, Ref= reference value, MAP= mean arterial pressure; Dry= dry mucus membranes and decreased skin turgor, Euvolemic = moist mucus membranes and normal jugular venous pressure, Wet= elevated jugular venous pressure and bilateral 1+ pitting edema; COPD= Chronic Obstructive Pulmonary Disease, ESRD= end-stage renal disease, HD= hemodialysis-dependent, HFrEF= heart failure with reduced ejection fraction; PIV= peripheral venous catheter, CVC= central venous catheter

© 2024 Munroe ES et al. *JAMA Network Open.*

© 2024 Munroe ES et al. *JAMA Network Open.*

eTable 2b. Association of randomized clinical factors with recommendations for fluids and vasopressors, Cases 3 and 4†

	Recommend Fluids			Recommend Vasopressors		
	Odds Ratio (95% CI)	Adjusted Proportion of Respondents* Percent (95% CI)	p-value	Odds Ratio (95% CI)	Adjusted Proportion of Respondents* Percent (95% CI)	p-value
**Fluid Volume**						
1 Liter	Ref.	82.8 (78.8, 86.9)		Ref.	55.4 (50.4, 60.3)	
2 Liters	0.10 (0.05, 2.00)	54.8 (49.5, 60.2)	<0.001	4.94 (2.93, 8.31)	77.7 (73.5, 82.0)	<0.001
5 Liters	0.002 (0.004, 0.007)	8.4 (5.5, 11.3)	<0.001	31.85 (14.20, 71.42)	92.3 (90.4, 95.6)	<0.001
**MAP (blood pressure)**						
52 mmHg (68/44)	2.51 (1.37, 4.57)	52.6 (48.5, 56.6)	0.003	9.44 (4.94, 18.05)	85.78 (82.3, 89.2)	<0.001
58 mmHg (74/48)	1.93 (1.07, 3.47)	50.0 (45.9, 54.1)	0.028	5.21 (2.95, 9.20)	80.29 (76.4, 84.2)	<0.001
64 mmHg (82/56)	Ref.	43.5 (39.2, 47.8)		Ref.	59.83 (55.1, 64.5)	
**Oxygen Status**						
50% Face Mask	0.48 (0.27, 0.86)	47.3 (43.0, 51.6)	0.014	1.65 (0.98, 2.78)	76.5 (72.4, 80.6)	0.059
6 Liters Nasal Cannula	0.37 (0.20, 0.67)	44.6 (40.5, 48.8)	0.001	1.94 (1.14, 3.29)	78.1 (74.3, 81.9)	0.014
Room Air	Ref.	54.4 (50.4, 58.4)		Ref.	70.9 (66.7, 75.1)	
**Respiratory Rate**						
20	Ref.	52.8 (48.7, 56.9)		Ref.	68.5 (64.2, 72.8)	
30	0.60 (0.34, 1.07)	47.8 (43.7, 51.9)	0.084	2.05 (1.23, 3.41)	76.6 (72.7, 80.6)	0.006
40	0.47 (0.26, 0.86)	45.5 (41.2, 49.7)	0.015	3.00 (1.73, 5.21)	80.5 (76.7, 84.2)	<0.001
**Case**						
Case 3 (PIV)	0.71 (0.47, 1.08)	47.0 (43.7, 50.4)	0.110	0.66 (0.45, 0.97)	72.9 (69.7, 76.2)	0.036
Case 4 (PORT)	Ref.	50.4 (47.1, 53.6)		Ref.	77.4 (74.3, 80.5)	

e-Table 4b Legend: Association of randomized clinical factors with participant recommendation for fluids and vasopressors in cases 3 and 4. Odds ratios and adjusted proportion of respondents were determined using separate multivariable, multilevel logistic regression models for 1) the recommendation to prescribe fluid and 2) the recommendation to initiate vasopressors. Multilevel models allowed clustering by participant ID, which was treated as a random effect.
N= 1,045 completed vignettes.
†Corresponds to Figure 1b.
*Adjusted proportion of respondents (also known as average predicted probabilities) were calculated using predictive margins after fitting each regression model. These predicted probabilities represent the proportion of respondents (as a percentage) recommending fluids or vasopressors based on the listed factors, after adjusting for all other factors in the model.

Definitions: CI= confidence interval, Ref= reference value; MAP= mean arterial pressure, Respiratory rate levels: 20 breaths per minute and no accessory muscle use; 30 breaths per minute and mild accessory muscle use; 40 breaths per minute and notable accessory muscle use.

© 2024 Munroe ES et al. *JAMA Network Open.*

© 2024 Munroe ES et al. *JAMA Network Open.*

**eTable 2c.** Association of randomized clinical factors with recommendations for fluids and vasopressors, Cases 5 and 6†						
	Recommend Fluids			Recommend Vasopressors		
	Odds Ratio (95% CI)	Adjusted Proportion of Respondents* Percent (95% CI)	P-value	Odds Ratio (95% CI)	Adjusted Proportion of Respondents* Percent (95% CI)	P-value
**Fluid Volume**						
1 Liter	Ref.	91.6 (88.7, 94.6)		Ref.	42.7 (37.7, 47.6)	
2 Liters	0.19 (0.10, 0.36)	75.9 (71.3, 80.5)	<0.001	8.90 (5.03, 15.75)	77.1 (72.8, 81.3)	<0.001
5 Liters	0.009 (0.003, 0.023)	26.3 (21.6, 31.1)	<0.001	49.77 (21.36, 115.97)	92.5 (89.9, 95.2)	<0.001
**MAP (blood pressure)**						
52 mmHg (68/44)	1.13 (0.68, 1.90)	66.0 (61.8, 70.2)	0.631	8.10 (4.43, 14.81)	80.7 (77.1, 84.4)	<0.001
58 mmHg (74/48)	0.92 (0.56, 1.53)	63.7 (59.6, 67.9)	0.758	5.37 (3.10, 9.31)	76.4 (72.4, 80.4)	<0.001
64 mmHg (82/56)	Ref.	64.6 (60.5, 68.7)		Ref.	54.7 (50.1, 59.2)	
**Lactate**						
Decreasing	1.16 (0.70, 1.93)	64.8 (60.7, 67.0)	0.558	1.06 (0.67, 1.68)	68.7 (64.5, 72.8)	0.805
Increasing	1.32 (0.79, 2.20)	66.2 (62.1, 70.3)	0.284	2.10 (1.27, 3.47)	76.6 (72.7, 80.5)	0.004
Stable	Ref.	63.2 (59.0, 67.3)		Ref.	67.9 (63.7, 72.2)	
**Acute Kidney Injury (AKI)**						
No AKI	Ref.	63.0 (58.8, 67.1)		Ref.	66.6 (62.3, 70.9)	
Non-oliguric AKI	1.16 (0.69, 1.94)	64.6 (60.4, 68.9)	0.568	1.90 (1.16, 3.09)	75.2 (70.3, 78.2)	0.010
Oliguric AKI	1.40 (0.84, 2.32)	66.6 (62.6, 70.7)	0.201	1.55 (0.97, 2.49)	71.9 (67.9, 76.0)	0.069
**Case**						
Case 5 (PIV)	0.86 (0.59, 1.25)	63.9 (60.6, 67.2)	0.418	0.82 (0.57, 1.18)	69.8 (66.5, 73.1)	0.281
Case 6 (PICC)	Ref.	65.6 (62.3, 68.9)		Ref.	72.2 (68.9, 75.4)	

e-Table 4c Legend: Association of randomized clinical factors with participant recommendation for fluids and vasopressors in cases 5 and 6. Odds ratios and adjusted proportion of respondents were determined using separate multivariable, multilevel logistic regression models for 1) the recommendation to prescribe fluid and 2) the recommendation to initiate vasopressors. Multilevel models were performed to allow clustering by participant ID, which was treated as a random effect.
N=1,041 completed vignettes.
†Corresponds to Figure 1c.
*Adjusted proportion of respondents (also known as average predicted probabilities) were calculated using predictive margins after fitting each regression model. These predicted probabilities represent the proportion of respondents (as a percentage) recommending fluids or vasopressors based on the listed factors, after adjusting for all other factors in the model.

Definitions: OR= odds ratio, Ref= reference value; MAP= mean arterial pressure, Lactate decreasing= initial lactate 4.1 mmol/L decreased to 2.7 mmol/L with fluids, Lactate repeat pending= initial lactate 4.1 mmol/L with repeat pending, Lactate increasing= initial lactate 4.1 mmol/L increased to 5.4mmol/L despite fluids; PICC=peripherally-inserted central catheter

© 2024 Munroe ES et al. *JAMA Network Open.*

eTable 3. Characteristics of Survey Participants Compared to Society of Critical Care Medicine (SCCM) Providers Who Received the Survey

Characteristics	Participants (N=550), No (%)	All SCCM members (N=11,203), No (%)	p-value*
**Gender**			
Male	261 (57.6%)	3,354 (60.7%)	0.190
Female	192 (42.4%)	2,167 (39.3%)	
**Clinical role**			
Physician	337 (71.7%)	8804 (78.6%)	
APP	101 (21.5%)	2,392 (21.4%)	<0.001
Other	32 (6.8%)	6 (0.05%)	
**Region of practice**			
Northeast	141 (30.0%)	2823 (26.3%)	
Midwest	121 (25.7%)	2601 (24.2%)	
South	118 (25.1%)	3397 (31.6%)	0.052
West	88 (18.7%)	1867 (17.4%)	
Outside United States	2 (0.4%)	54 (0.5%)	

Legend: Comparison of participant characteristics to characteristics of SCCM members who were sent the electronic survey. SCCM member demographics were provided by SCCM. N=550 providers completed the first clinical vignette of this survey and were considered study participants. Information was missing or not reported for gender (N=97), clinical role (N=80), region of practice (N=80). The survey was sent to 11,203 SCCM members (US-based critical care providers). Clinical role was available for all SCCM members; information was missing for gender (N=5,681) and region of practice (N=46).

*p-values were determined using Chi-Squared tests of difference. Comparisons were made after excluding missing information.

eFigure 1. Perceived Realism of Clinical Vignettes

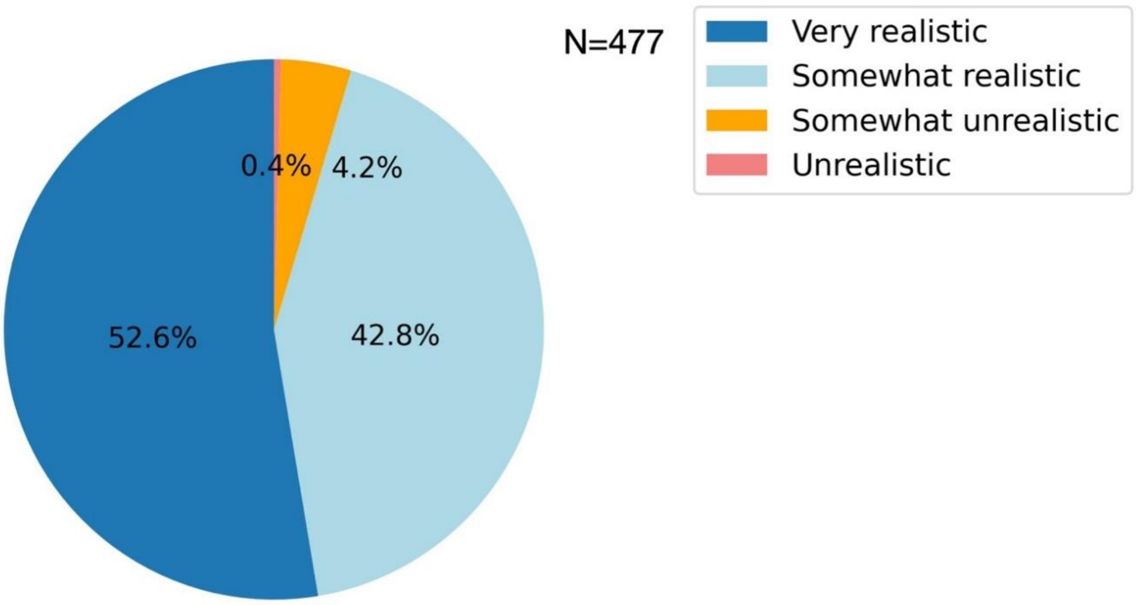

© 2024 Munroe ES et al. *JAMA Network Open.*

eFigure 2. Self-Reported Usual Practices For Managing Patients With New Sepsis-Induced Hypotension

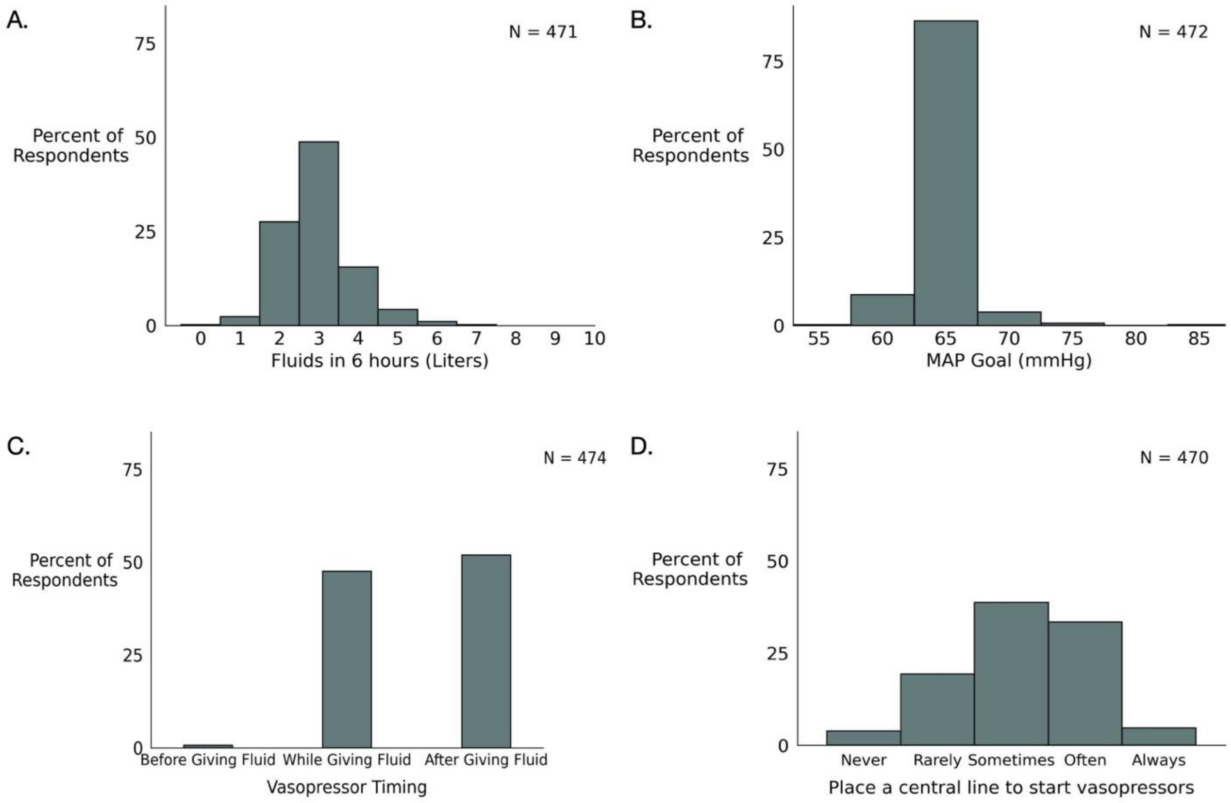

<u>e-Figure 2 Legend.</u> Respondents were asked to report their usual (average) practices for managing patients with new sepsis-induced hypotension. A. Amount of IV fluid given within the first 6 hours of patient presentation, in liters. B. Mean Arterial Pressure (MAP) goal, in mmHg. C. Timing of vasopressor initiation for patients with persistent hypotension. D. Route of vasopressor initiation.

© 2024 Munroe ES et al. *JAMA Network Open.*

eFigure 3. Range of Respondent Answers Across Cases

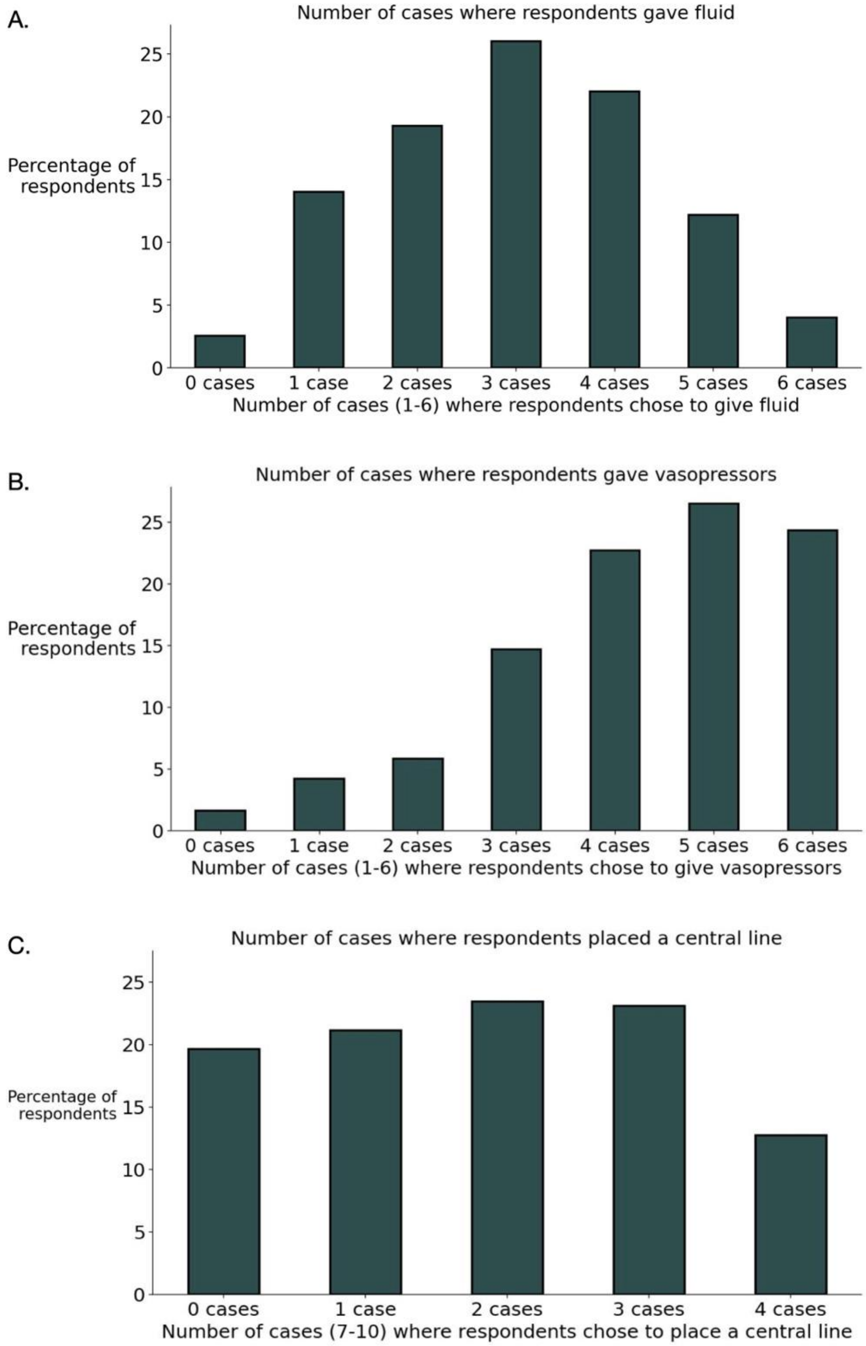

<u>eFigure 3 Legend</u>: This figure depicts the range respondent answers across cases. Making the same choice in none (0) or all cases may reflect that a respondent has a set practice that they were not changing based on factors in the case. In contrast, choosing a response in only some of the cases (e.g., 3/6 cases) may indicate that the respondent was personalizing care—or changing their response—based on the factors presented in the cases.

© 2024 Munroe ES et al. *JAMA Network Open.*

eFigure 4. Range of Respondent Answers Across Cases Where Participants Saw the Same Fluid Volume Already Received

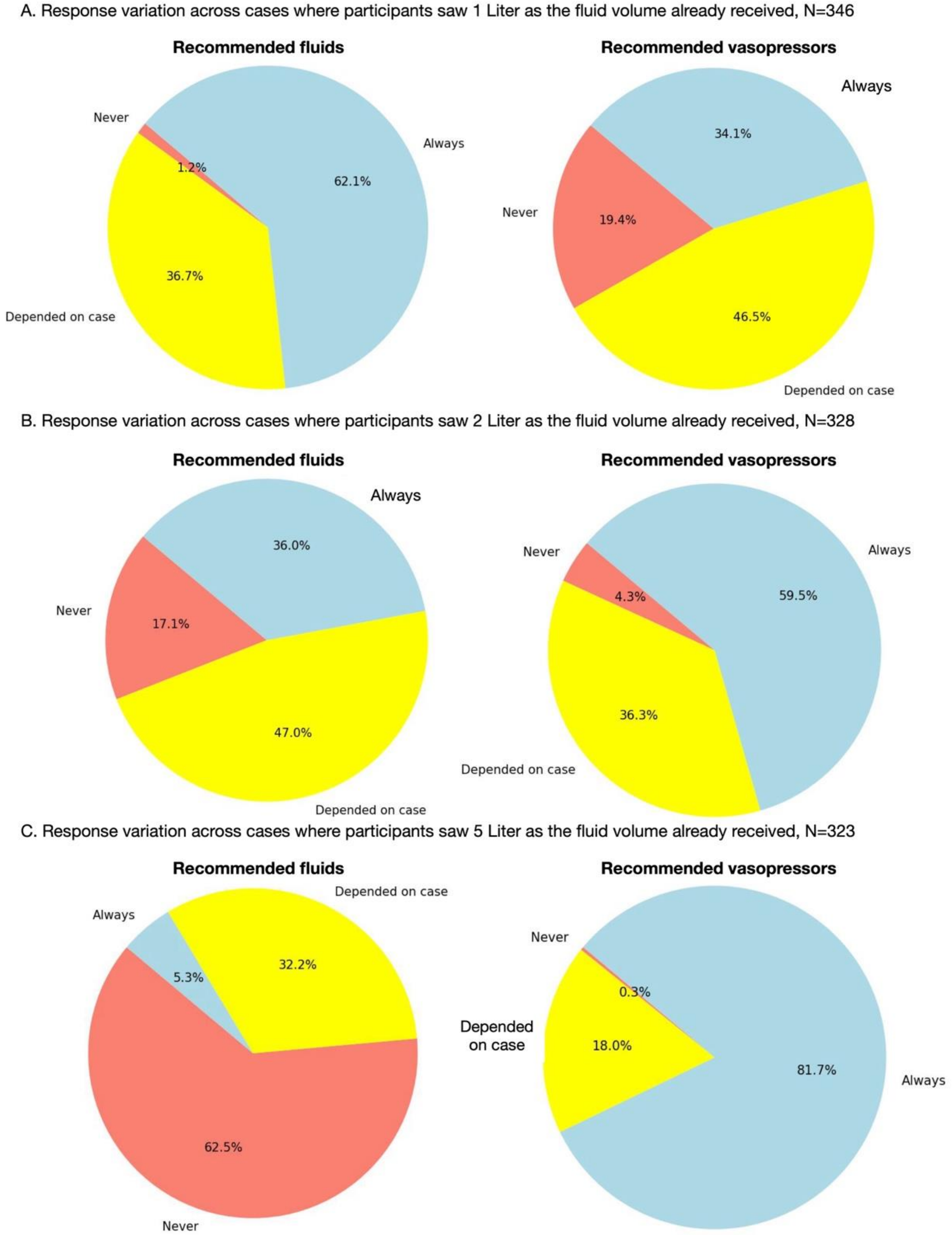

<u>eFigure 4 Legend</u>: This figure depicts the variation in respondent recommendations for fluids and vasopressors across cases where respondents saw the same fluid volume volume already received. Respondents were included if they saw the same fluid volume already received in ≥ 2 cases (e.g., saw 2 or more cases where the fluid volume already received was 1L, in panel A). Red and blue represent the percent of respondents who always made the same recommendations in cases with the same fluid volume already received, either never (red) or always (blue) making a recommendation. Yellow represents the number of respondents who made different recommendations across cases where they saw the same fluid volume already received (e.g., started fluids in only some of the cases they saw with the same fluid volume). This yellow portion reflects the percent of respondents whose decisions appeared to depend on other factors in the case.

© 2024 Munroe ES et al. *JAMA Network Open.*

**eTable 4.** Association of Fluid Volume, MAP, and Case With Perceived Case Difficulty, Cases 1-6				
	Odds Ratio of a difficult decision†	95% CI	P-value	Adjusted proportion of respondents* Percent (95% CI)
**Fluid Volume**				
1 Liter	Ref.			9.6 (7.4, 11.8)
2 Liters	1.08	(0.73, 1.61)	0.686	10.1 (7.8, 12.3)
5 Liters	0.76	(0.50, 1.16)	0.203	8.2 (6.2, 10.2)
**MAP (blood pressure)**				
52 mmHg (68/44)	0.92	(0.61, 1.37)	0.668	9.0 (7.0, 11.1)
58 mmHg (74/48)	0.96	(0.64, 1.44)	0.833	9.3 (7.2, 11.4)
64 mmHg (82/56)	Ref.			9.5 (7.3, 11.7)
**Case**				
1	1.46	(0.89, 2.38)	0.133	13.1 (10.1, 16.1)
2	Ref**			10.5 (7.8, 13.3)
3	0.72	(0.42, 1.23)	0.234	8.7 (6.2, 11.2)
4	0.71	(0.2, 1.20)	0.200	7.0 (4.8, 9.3)
5	0.51	(0.29, 0.90)	0.019	7.0 (4.8, 9.3)
6	0.60	(0.35, 1.03)	0.065	7.7 (5.4, 10.1)

e-Table 2 Legend: Association of fluid volume, MAP and case with perceived case difficulty assessed using a multivariable, multilevel logistic regression for difficult decision. In the multilevel model, responses were clustered by participant. N=3,097 completed vignettes

†Participants were asked to rate the difficulty of each on a five-point Likert scale. A difficult decision was defined as a response of "very difficult" and "somewhat difficult."

*Adjusted proportion of respondents (also known as average predicted probabilities) were calculated using predictive margins after fitting the regression model. These predicted probabilities represent the proportion of respondents (as a percentage) reporting a difficult decision based on the listed factors, after adjusting for all other factors in the model.

**Case 2 was selected as the reference case because the patient presented in case 2 had a temporary central venous catheter, the gold standard route for vasopressor administration. Patients presented in other cases had only peripheral IVs (cases 1,3,5), a pre-existing Port (case 4), or a pre-existing peripherally-inserted central catheter (case 6).

Definitions: MAP= Mean Arterial Pressure, CI= Confidence Interval

© 2024 Munroe ES et al. *JAMA Network Open.*

eFigure 5. Overall Association of Fluid Volume and MAP With Recommendations for Fluids and Vasopressors, Cases 1-6

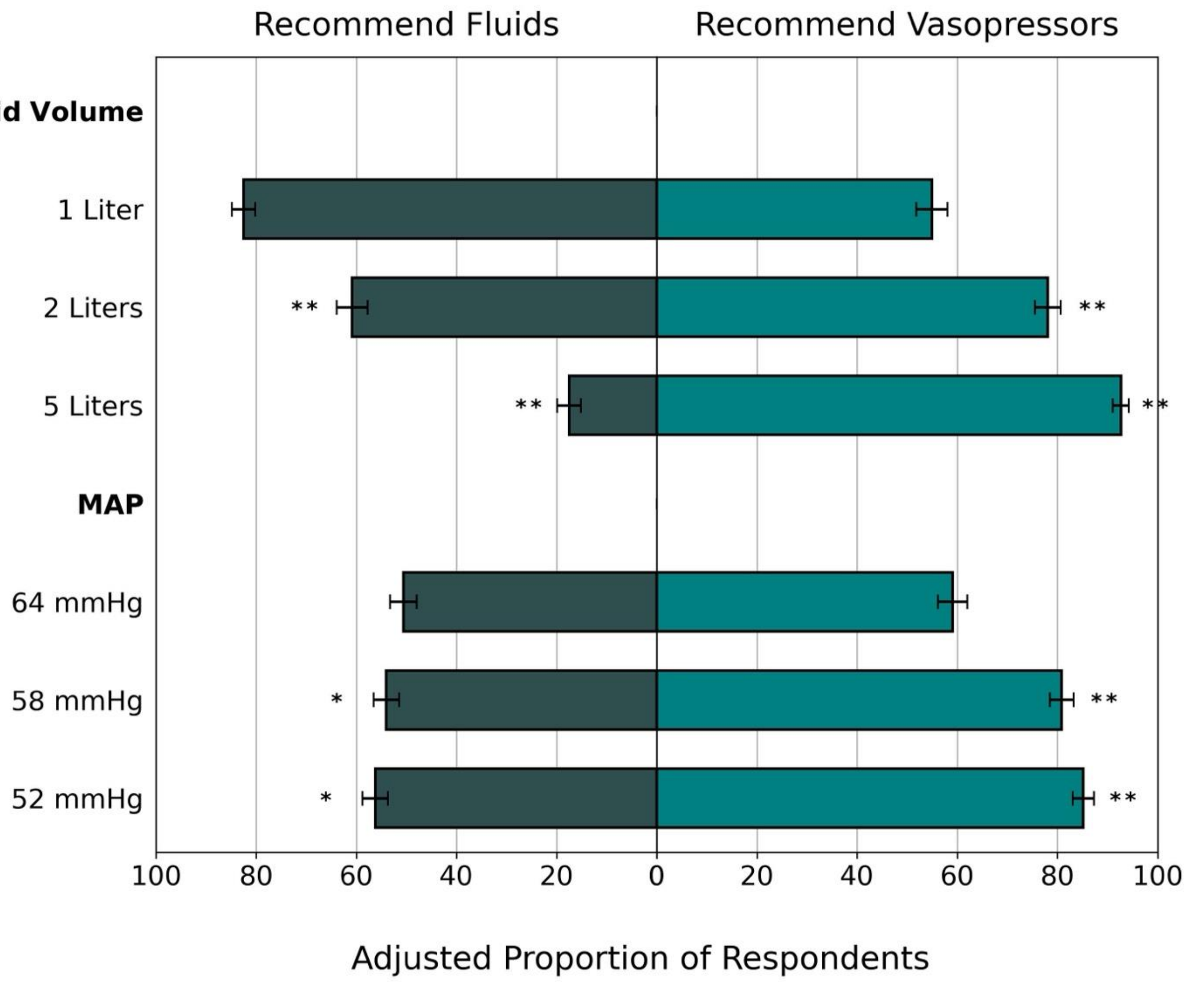

eFigure 5 Legend: Adjusted proportion of respondents recommending fluids or vasopressors by fluid volume and MAP in cases 1-6. Fluid volume and MAP were randomized across all cases 1-6 and their overall effects are reported here. The other clinical factors included in cases 1-6 (volume status exam, medical history, oxygen requirement, respiratory rate, lactate trend, and acute kidney injury) were randomized in 2 out of 6 cases but kept constant in the other cases (e.g., oxygen requirement was randomized in cases 3 and 4 but kept constant at room air in cases 1, 2, 5, 6). These clinical factors were included as co-variates in these regressions, but given they were not randomized in each case, their overall effects are not reported. Effects of all randomized clinical factors are reported in separate case-paired regressions (in e-Table 2 and Figure 2). N=3,129 completed vignettes. Error bars represent 95% CIs.
*= p-value <0.05
** = p-value <0.001

© 2024 Munroe ES et al. *JAMA Network Open.*

eTable 5. Additional Requested Factors for Fluid and Vasopressor Decisions, Cases 1-6

Requested Information	Number of participants requesting information (N)
**Ultrasonography**	
POC Cardiac	39
Formal Echocardiogram	6
POC Lung	5
**Additional Labs / Imaging**	
Lactate	12
Complete Laboratory Assessment	4
Acid / Base Assessment	4
Chest X-Ray	3
Other[a]	4
**Vitals Signs / Physical Exam**	
Weight	48
Passive Leg Raise	11
Input / Output	8
Capillary Refill	5
Heart Rate	5
Respiratory Rate	2
Other[b]	3
**Additional Clinical Information**	
Prior Echocardiogram Results	9
Date of Last Hemodialysis Session	5
Prior Responsiveness to IV Fluids	4
Baseline Vitals / Physical Exam	3
Administration of Intravenous Contrast	1
**Additional Hemodynamic Monitoring**	
Non-invasive Cardiac Output Monitor	10
Pulmonary Arterial Catheter	6
CVP / SvO2	4
Pulse Pressure Variation	1
**Intravenous Catheter / Medication Data**	
Intravenous Catheter Location	4
Vasopressor Dosage	1
Intravenous Access Age	1

Legend: This table depicts additional information that was requested by participants in free-text comment boxes associated with cases 1-6. N=189 participants requested additional information for at least one case. If a participant requested the same information for multiple cases, it was only recorded once.

[a] Creatinine (n=2), albumin (n=1), and arterial blood gas (n=1)

[b] Jugular venous distention (n=1) and assessment of mentation (n=2)

Definitions: POC = point of care; IV= intravenous; CVP = central venous pressure; SvO2 = mixed venous oxygen saturation

© 2024 Munroe ES et al. *JAMA Network Open.*

**eTable 6a**. Association of randomized clinical factors with peripheral vasopressor initiation, when baseline vascular access was a PIV[†]									
	Case 1			Case 3			Case 5		
	Odds Ratio (95% CI)	Adjusted Proportion of Respondents* Percent (95% CI)	p-value	Odds Ratio (95% CI)	Adjusted Proportion of Respondents* Percent (95% CI)	p-value	Odds Ratio (95% CI)	Adjusted Proportion of Respondents* Percent (95% CI)	p-value
**Fluid Volume**									
1 Liter	Ref	91.4 (84.2, 94.1)		Ref.	91.9 (86.7, 97.1)		Ref.	93.7 (88.3, 99.1)	
2 Liters	0.95 (0.40, 2.30)	91.0 (81.5, 92.9)	0.917	1.69 (0.58, 4.94)	95.0 (91.1, 98.9)	0.339	0.65 (0.21, 2.01)	90.6 (85.3, 96.0)	0.453
5 Liters	0.59 (0.26, 1.33)	86.3 (87.7, 97.6)	0.203	0.63 (0.27, 1.51)	87.8 (82.7, 93.0)	0.301	0.79 (0.26, 2.41)	92.1 (87.5, 96.8)	0.674
**MAP (blood pressure)**									
52 mmHg (68/44)	0.65 (0.26, 1.61)	89.2 (84.2, 94.1)	0.350	0.87 (0.35, 2.20)	89.9 (84.8, 94.9)	0.772	0.51 (0.17, 1.46)	89.5 (84.4, 94.6)	0.205
58 mmHg (74/48)	0.54 (0.22, 1.31)	87.1 (81.5, 92.9)	0.170	1.24 (0.47, 3.30)	92.6 (88.3, 96.9)	0.663	0.75 (0.23, 2.49)	92.7 (88.0, 97.4)	0.638
64 mmHg (82/56)	Ref	92.7 (87.7, 97.6)		Ref.	91.0 (85.4, 96.7)		Ref.	94.4 (89.6, 99.3)	
**Volume status**									
Dry	0.64 (0.27, 1.51)	89.2 (83.8, 94.7)	0.305						
Wet	0.48 (0.21, 1.09)	86.2 (80.3, 92.0)	0.078						
Euvolemic	Ref	92.8 (88.6, 97.1)							
**Medical History**									
COPD	Ref	89.7 (84.5, 95.0)							
ESRD on HD	0.84 (0.38, 1.86)	88.1 (82.6, 93.5)	0.673						
HFrEF	1.08 (0.48, 2.34)	90.4 (85.6, 95.1)	0.857						
**Oxygen Status**									
50% Face Mask				1.87 (0.66, 5.32)	95.3 (91.7, 98.9)	0.241			
6 Liters NC				0.57 (0.24, 1.33)	86.3 (80.3, 92.4)	0.191			
Room Air				Ref.	91.7 (86.7, 96.6)				
**Respiratory Rate**									
20				Ref.	92.7 (88.0, 97.5)				
30				0.89 (0.33, 2.45)	92.0 (87.0, 96.9)	0.827			
40				0.65 (0.27, 1.61)	89.4 (84.7, 94.1)	0.353			
**Lactate**									
Decreasing							1.03 (0.37, 2.89)	92.0 (86.8, 97.2)	0.953
Increasing							1.04 (0.40, 2.71)	92.0 (87.2, 96.8)	0.942
Stable							Ref.	91.8 (86.5, 97.1)	
**Acute Kidney Injury (AKI)**									
No AKI							Ref.	91.0 (85.6, 96.3)	
Non-oliguric AKI							1.53 (0.55, 4.21)	93.9 (89.5, 98.3)	0.414
Oliguric AKI							1.00 (0.40, 2.46)	90.9 (85.9, 96.0)	0.991

Legend: Association of randomized clinical factors with peripheral vasopressor initiation in case 1, 3, and 5, where case patients had only 2 peripheral IVs as vascular access.
[†]In cases 1, 3, and 5, patients only had peripheral IVs as their baseline vascular access. Case 1: N= 406, Case 3: N=374, Case 5: N= 347. Participants only answered this question if they recommended vasopressors in the case. *Adjusted proportion of respondents (also known as average predicted probabilities) were calculated using predictive margins after fitting each regression model. These predicted probabilities represent the proportion of respondents (as a percentage) starting vasopressors peripherally (PIV only or PIV as a bridge to central access) based on the listed factors, after adjusting for all other factors in the model. *Definitions:* PIV= peripheral IV, Ref= reference value; CI= confidence interval; MAP= mean arterial pressure; Dry= dry mucus membranes and decreased skin turgor, Euvolemic = moist mucus membranes and normal jugular venous pressure, Wet= elevated jugular venous pressure and bilateral 1+ pitting edema; COPD= Chronic Obstructive Pulmonary Disease, ESRD= end-stage renal disease, HD= hemodialysis-dependent, HFrEF= heart failure with reduced ejection fraction; NC= nasal cannula, Respiratory rate levels: 20 breaths per minute and no accessory muscle use; 30 breaths per minute and mild accessory muscle use,40 breaths per minute and notable accessory muscle use; Lactate decreasing= initial lactate 4.1 mmol/L decreased to 2.7 mmol/L with fluids, Lactate repeat pending= initial lactate4.1 mmol/L with repeat pending, Lactate increasing= initial lactate 4.1 mmol/L increased to 5.4mmol/L despite fluids; PICC=peripherally-inserted central catheter

© 2024 Munroe ES et al. *JAMA Network Open.*

**eTable 6b**. Association of randomized clinical factors with peripheral vasopressor initiation, when baseline vascular access was a pre-existing central catheter[†]						
	Pre-existing PORT (Case 4)			Pre-existing PICC (Case 6)		
	Odds Ratio (95% CI)	Adjusted Proportion of Respondents* Percent (95% CI)	p-value	Odds Ratio (95% CI)	Adjusted Proportion of Respondents* Percent (95% CI)	p-value
**Fluid Volume**						
1L	Ref.	32.3 (22.8, 41.8)		Ref.	26.7 (12.2, 37.2)	
2L	0.97 (0.54, 1.72)	31.6 (23.7, 39.4)	0.903	0.63 (0.32, 1.27)	18.7 (12.2, 25.3)	0.195
5L	0.86 (0.49, 1.51)	29.2 (22.0, 36.4)	0.604	0.42 (0.20, 0.84)	13.1 (7.7, 18.4)	0.015
**MAP**						
68/44 (52)	0.91 (0.52, 1.60)	29.6 (22.2, 37.0)	0.754	0.89 (0.45, 1.77)	17.4 (11.1, 23.8)	0.735
74/48 (58)	1.00 (0.57, 1.78)	31.6 (23.7, 39.4)	0.988	0.91 (0.46, 1.79)	17.7 (11.3, 24.1)	0.774
82/56 (64)	Ref.	31.5 (22.5, 40.5)		Ref.	19.2 (11.3, 27.1)	
**Oxygen Status**						
50% Face Mask	0.75 (0.44, 1.29)	27.7 (20.1, 35.3)	0.301			
6 Liters NC	0.88 (0.52, 1.50)	31.1 (23.3, 38.8)	0.644			
Room Air	Ref.	33.7 (25.3, 42.2)				
**Respiratory Rate**						
20	Ref.	32.7 (24.3, 41.1)				
30	0.65 (0.37, 1.14)	24.0 (16.7, 31.2)	0.131			
40	1.16 (0.69, 1.97)	36.1 (27.9, 44.4)	0.572			
**Lactate**						
Decreasing				1.25 (0.62, 2.52)	19.6 (12.3, 26.9)	0.535
Increasing				1.11 (0.57, 2.19)	17.9 (11.3, 24.4)	0.758
Stable				Ref.	16.4 (9.7, 23.1)	
**AKI**						
No AKI				Ref.	18.4 (11.2, 25.6)	
Non-oliguric AKI				0.98 (0.50, 1.92)	18.1 (11.5, 24.7)	0.956
Oliguric AKI				0.94 (0.48, 1.84)	17.5 (10.9. 24.0)	0.851

Legend: Association of randomized clinical factors with peripheral vasopressor initiation in case 4 (baseline access=Port) and case 6 (baseline access=PICC)

[†]In case 4, the patient had a pre-existing PORT (N= 390). In case 6, the patient had a pre-existing PICC (N=367). Case 2 was not included in this analysis because the patient in case 2 had a pre-existing new temporary central line, which was presumed to be the default route of vasopressor initiation. Participants only answered this question if they recommended vasopressors in the case.

*Adjusted proportion of respondents (also known as average predicted probabilities) were calculated using predictive margins after fitting each regression model. These predicted probabilities represent the proportion of respondents (as a percentage) starting vasopressors peripherally (PIV only or PIV as a bridge to central access) based on the listed factors, after adjusting for all other factors in the model.

Definitions: PIV= peripheral IV, PICC=peripherally-inserted central catheter, CI=Confidence Interval, Ref= reference value; MAP= mean arterial pressure; NC= nasal cannula, Respiratory rate levels: 20 breaths per minute and no accessory muscle use; 30 breaths per minute and mild accessory muscle use,40 breaths per minute and notable accessory muscle use; AKI= Acute Kidney Injury, Lactate decreasing= initial lactate 4.1 mmol/L decreased to 2.7 mmol/L with fluids, Lactate repeat pending= initial located 4.1 mmol/L with repeat pending, Lactate increasing= initial lactated 4.1 mmol/L increased to 5.4mmol/L despite fluids.

© 2024 Munroe ES et al. *JAMA Network Open.*

eTable 7. Association of Randomized Clinical Factors With Recommendation to Place a Central Line in Patients Receiving Peripheral Vasopressors, Cases 7-10⊥

	Place a Central Line			
	Odds Ratio	95% CI	p-value	Adjusted Proportion of Respondents* Percent (95% CI)
**Norepinephrine dose**				
0.08 mcg/kg/min	Ref.			25.2 (21.8, 28.5)
0.2 mcg/kg/min	10.90	(7.29, 16.31)	<0.001	56.6 (52.7, 60.4)
0.5 mg/kg/min	61.67	(36.87, 103.15)	<0.001	78.0 (74.7, 81.2)
**Vasopressor trend**				
Stable	Ref.			52.3 (48.7, 55.9)
Decreasing	0.27	(0.19, 0.40)	<0.001	36.3 (32.8, 39.9)
Increasing	4.91	(3.37, 7.16)	<0.001	71.0 (67.7, 74.2)
**Duration**				
8 hours	Ref			47.1 (44.0, 50.1)
24 hours	2.87	(2.12, 3.90)	<0.001	59.5 (56.6, 62.5)
**PIV Location†**				
Upper Arm	Ref			52.8 (49.9, 55.8)
Forearm	1.08	(0.81, 1.45)	0.589	53.7 (50.7, 56.7)
**Case**				
Case 7	Ref			54.1 (50.4, 57.7)
Case 8	0.85	(0.58, 1.25)	0.407	52.2 (48.5, 55.9)
Case 9	0.76	(0.52, 1.11)	0.159	50.9 (47.2, 54.6)
Case 10	1.18	(0.81, 1.72)	0.381	56.0 (52.4, 59.6)

e-Table 7 Legend: Association of randomized factors with the recommendation to place a central line in patients already receiving peripheral norepinephrine (cases 7-10). Patients presented in these cases were all receiving norepinephrine through an 18-gauge peripheral IV. Respondents were asked if they would continue the vasopressor peripherally or place central access (temporary central venous catheter or peripherally-inserted peripheral catheter). A multivariable, multilevel logistic regression model was performed to allow clustering by participant ID, which was treated as a random effect. N=1,936 vignettes.

⊥ Corresponds to Figure 3

*Adjusted proportion of respondents (also known as average predicted probabilities) were calculated using predictive margins after fitting each regression model. These predicted probabilities represent the proportion of respondents (as a percentage) recommending placing a central line based on the listed factors, after adjusting for all other factors in the model.

†PIV location excluded the antecubital fossa.

Definitions: CI= confidence interval, Low dose= 0.08 mcg/kg/min (5 mcg/min) norepinephrine, Medium dose= 0.20 mcg/kg/min (15 mcg/min) norepinephrine, High dose= 0.50 mcg/kg/min (35 mcg/min) norepinephrine, PIV= peripheral IV

Case details: Case 7= 55 female with cholecystitis, Case 8= 70 male with pneumonia, Case 9= 65 female with urosepsis, Case 10= 60 male with cellulitis

© 2024 Munroe ES et al. *JAMA Network Open.*

eTable 8. Identified Themes From a Free-Text Question Eliciting Factors That Contributed to Respondents' Decision to Place a Central Line in Patients on Peripheral Vasopressors, Cases 7-10

**Important Factor**	**Frequency (N participants)**
**Vasopressor Administration Data**	
Dosage Trend and Clinical Trajectory	18
Current Dosage	4
Total Length of Administration	3
**Plans for Anticipated Procedures / Surgeries**	7
**Available Hospital Resources**[a]	4
**Hospital Policy**	4
**Other Medications or Monitoring Requiring Central Access**	4
**Adequacy of Fluid Resuscitation**	2
**Renal Function**	1

Legend: This table displays the themes identified by reviewing responses.In free-text comments, N=33 respondents commented on additional information they would use to make decisions about central line placement in cases 7-10. These themes represent factors that participants reported using when making decisions about when to place central lines in patients on peripheral vasopressors.

[a]For example, availability of a team capable of placing a peripherally-inserted central catheter (PICC), "nursing capabilities"

eFigure 6. Self-Reported Factor That Most Influences the Decision to Use Peripheral Vasopressors

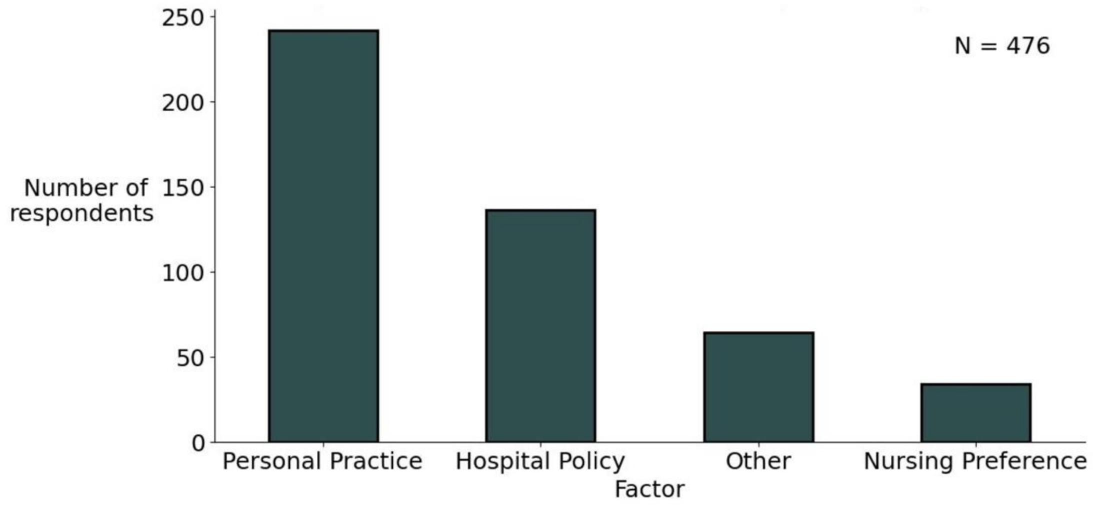

© 2024 Munroe ES et al. *JAMA Network Open.*

eTable 9. Identified Themes From a Free-Text Question About Most Important Factors Impacting Decision About Peripheral Vasopressor Use

**Other Factor**	**Frequency of other factor (N participants)**
**Vasopressor Administration Data**	
Dosage Trend and Clinical Trajectory	25
Current Dosage	17
Total Length of Administration	12
**PIV Access Concerns**	
Inadequate Peripheral Venous Access[a]	22
Location / Perceived Quality of PIV Access	6
**Availability of Hospital Resources[b]**	7
**Hospital Policy**	3
**Adequacy of fluid resuscitation**	3
**Risks of Central Venous Catheter Placement**	2
**Urgency of Vasopressor Initiation**	2
**Prior Experience with Peripheral Vasopressor Complications**	1
**Lactate**	1

Legend: Providers were asked "In your practice, for patients whose only indication for central access is vasopressor infusion, what factor most influences your decision to place a central line?' Answer options were: hospital policy, nursing preference, personal practice, and "other," with an associated free-text box. Here we present themes identified from review of free-text "other" responses (N=63 participants).

[a]For example, central access needed for other medications, medication incompatibility, lab draws, or CVP/SvO2 monitoring; inability to attain peripheral intravenous access.
[b]For example, "business of the unit"; time of day; physician availability; availability of a team capable of placing a peripherally-inserted central catheter (PICC); availability of subspeciality care.

Definitions: PIV= peripheral intravenous catheter

© 2024 Munroe ES et al. *JAMA Network Open.*
