# Supplementary material for: Understanding How Clinicians Personalize Fluid and Vasopressor Decisions in Early Sepsis Management
Source: JAMA Netw Open. 2024 Apr 19;7(4):e247480. doi: 10.1001/jamanetworkopen.2024.7480 (PMC11031682; doi:10.1001/jamanetworkopen.2024.7480)
Supplement: Supplement 2. — Data Sharing Statement [file jamanetwopen-e247480-s002.pdf]

Data Sharing Statement

Munroe. Understanding How Clinicians Personalize Fluid and Vasopressor Decisions in Early Sepsis Management. *JAMA Netw Open*. Published April 19, 2024.
doi:10.1001/jamanetworkopen.2024.7480

Data
Data available: Yes
Data types: Deidentified participant data
How to access data: Requests for data can be sent to munroeel@med.umich.edu
When available: With publication

Supporting Documents
Document types: Other (please specify)
Additional Information: Full Survey
How to access documents: Online Supplement
When available: With publication

Additional Information
Who can access the data: anyone requesting the data
Types of analyses: for any purpose
Mechanisms of data availability: with investigator support
